# Integrating Kinetic Model of *E*. *coli* with Genome Scale Metabolic Fluxes Overcomes Its Open System Problem and Reveals Bistability in Central Metabolism

**DOI:** 10.1371/journal.pone.0139507

**Published:** 2015-10-15

**Authors:** Ahmad A. Mannan, Yoshihiro Toya, Kazuyuki Shimizu, Johnjoe McFadden, Andrzej M. Kierzek, Andrea Rocco

**Affiliations:** 1 Faculty of Health and Medical Sciences, University of Surrey, Guildford, Surrey, GU2 7XH, United Kingdom; 2 Department of Bioinformatic Engineering, Graduate School of Information Science and Technology, Osaka University, 1–5 Yamadaoka, Suita, Osaka, 565–0871, Japan; 3 Institute for Advanced Biosciences, Keio University, Nipponkoku 403–1, Daihouji, Tsuruoka, Yamagata, 997–0017, Japan; 4 Department of Bioscience and Bioinformatics, Kyushu Institute of Technology, 680–4 Kawazu, Iizuka-shi, Fukuoka, 820–8502, Japan; University of Nebraska Medical Center, UNITED STATES

## Abstract

An understanding of the dynamics of the metabolic profile of a bacterial cell is sought from a dynamical systems analysis of kinetic models. This modelling formalism relies on a deterministic mathematical description of enzyme kinetics and their metabolite regulation. However, it is severely impeded by the lack of available kinetic information, limiting the size of the system that can be modelled. Furthermore, the subsystem of the metabolic network whose dynamics can be modelled is faced with three problems: how to parameterize the model with mostly incomplete steady state data, how to close what is now an inherently open system, and how to account for the impact on growth. In this study we address these challenges of kinetic modelling by capitalizing on multi-*‘omics’* steady state data and a genome-scale metabolic network model. We use these to generate parameters that integrate knowledge embedded in the genome-scale metabolic network model, into the most comprehensive kinetic model of the central carbon metabolism of *E*. *coli* realized to date. As an application, we performed a dynamical systems analysis of the resulting enriched model. This revealed bistability of the central carbon metabolism and thus its potential to express two distinct metabolic states. Furthermore, since our model-informing technique ensures both stable states are constrained by the same thermodynamically feasible steady state growth rate, the ensuing bistability represents a temporal coexistence of the two states, and by extension, reveals the emergence of a phenotypically heterogeneous population.

## Introduction

In this era of mass information, advancing technologies exploited in molecular biology research are enabling high throughput generation of multiple types of *‘-omics’* data. This is constantly fuelling the interests of biologists to view and understand the functioning of living cells as an integrated system of molecular interaction networks [1,2]. Construction of a mathematical model formalises the description of these networks quantitatively. This also provides a framework for the integration of data and the application of engineering techniques and mathematical analyses to understand the control of different components on the cellular system [1,3].Ultimately, this enables the prediction of emergent cellular behaviours.

Metabolism drives the functioning and growth of a cell through a highly complex network of biochemical interactions, converting nutrients taken up into energy, cellular building blocks and signalling molecules. A description of the metabolite composition of the cell can thus be used to characterise it phenotype at a given time point during growth, given nutrient availability and growth conditions. An understanding of the dynamical response of the cell to changes in nutrient availability and how these shift its metabolic states, phenotypic profile, and thus alter cell behaviour, has received much attention from the perspective of mathematical modelling, particularly of bacterial metabolism [4–12].

Bacteria play a vital role in many globally important chemical cycles, such as the nitrogen cycle, and are of enormous importance in both biotechnology and medicine. In biotechnology, they are often employed as a more efficient means of producing biochemical products of metabolism [4]. In medicine they are encountered as components of the normal flora of man and animals as well as being responsible for major diseases that kill millions a year [5–7]. Modelling the dynamical response and metabolic shift of the bacterial cell is therefore crucial to gaining an understanding of how they persist in the environment and cause disease, as well as how they can be optimized for biotechnological production.

One of two principal approaches is usually adopted for the modelling. In the first approach, a genome-scale metabolic network (GSMN) model is constructed that captures the stoichiometry of all known metabolic conversions in the cell. GSMN models can be used to make predictions of reaction flux rates, cell growth rate and product production rates, as well as to predict gene essentiality, helping to identify drug targets at the genome scale [5,7]. However, these models can only be used to describe the cell metabolism at steady state, and their application to real world systems is therefore limited [2].

In the second approach, a kinetic model of the biochemical reactions representing the cell metabolism is constructed to simulate the dynamical behaviour of metabolite concentrations and reaction fluxes. This model incorporates the enzyme kinetics of every reaction within the metabolic network in a deterministic fashion, likes the models of [4,8,9,13].

To make precise quantitative predictions of the metabolic state of the cell and of its growth phenotype, both at steady state and during dynamical growth, one can envision the construction of a genome scale kinetic model [2]. However, progression towards this goal faces a number of fundamental problems. These include the severe lack in knowledge of the reaction enzyme kinetics on the genome scale, incomplete knowledge of the kinetic parameters, and the non-availability of steady state reaction flux and metabolite concentration values. Missing steady state data exasperates the determination of kinetic parameter values as it results in a mathematically ill-posed problem for parameter determination. These problems severely limit the size of the metabolic network that can be modelled dynamically.

A sufficiently well characterized subset of the full network is the central carbon metabolism. The criticality of this set of reactions for the production of energy and biosynthetic precursors have made them the focus of many studies [4,8–12,14]. However, modelling the dynamics of only this subnetwork brings two further problems into play. Firstly, how to close what becomes an inherently open system. In particular, one cannot account for the contribution back to and from the rest of metabolism to the subnetwork modelled. This in turn brings about a second issue, the ability to account directly for the impact onto growth rate.

In this study we show how these two fundamental problems of kinetic modelling can be addressed by integrating a novel kinetic model of the central carbon metabolism of the model bacterium *Escherichia coli* with steady state data. The steady state data used were taken from the Keio multi-omics dataset [15,16], which reports various omics measurements from a particular steady state culture experiments. This means that all data used are coming from the same source and thus are consistent with one another in representing the cell state under the same growth and physical conditions. These include a large number of measurements of the culture fluxome, metabolome, proteome and transcriptome. In particular we show how the integration of the flux data, determined from a GSMN model of *E*. *coli*, both closes the ‘open system’ problem of the kinetic model and ensures a direct and thermodynamically feasible account of the specific cell growth rate.

The model resulting from the integration of the steady state fluxes can be used to understand the cellular metabolic steady states during steady state growth conditions. This would be equivalent to observing cell and population growth during the constant growth rate of the exponential growth phase. Since we are focusing on the metabolic state of the cell, we are making the assumption that gene regulation and translation (enzyme production) is at quasi-steady state. It is critical to realize that the dynamics and metabolic states discovered from the mathematical analysis of our kinetic model are therefore valid only on a time scale shorter than the time scales characterizing the full dynamics of changes in the cell.

In this study we demonstrate an application of the constructed model to understand cellular behaviour. Recent studies into the emergence of alternative phenotypes have elucidated the coexistence of two distinct phenotypes in an isogenic population [17–19], even under steady state growth conditions [20,21]. Though both phenotypes can coexist, they are observed to grow at different growth rates, with one expressing an impaired growth. This switch in growth phenotype becomes apparent after a substrate shift, where the phenotypic profile of the population prior to the substrate perturbation is assumed homogeneous [17,18]. An interesting question emerges from this observation as to whether alternative metabolic phenotypes existed prior to the media perturbation and subsequent change in growth rates. We hypothesize that the pre-existence of alternative states of the metabolic system would then give rise to the phenotypic heterogeneity that is observed on the longer time scale of the cell dynamics. In this study we address this question and our hypothesis by using a novel and detailed kinetic model. In particular, we ask whether we can find alternative stable steady states of the central carbon metabolism for both a fixed media condition and a fixed growth rate. To answer this question, we incorporated both these constraints into the kinetic model to simulate the fixed growth conditions before substrate shift, and then performed a dynamical systems analysis of the model. Our analysis did in fact reveal two stable steady states of the central metabolism. Consideration of the change in metabolite profile between these two states allowed us to hypothesize about the consequential change in cell phenotype. Namely, we hypothesize that one metabolic state corresponds to a cell consuming glucose, while the other is geared to converge to a phenotype defined by its consumption of acetate. This means that we hypothesize the emergence of two coexisting subpopulations in steady state conditions, one subpopulation consuming glucose, while the other would consume acetate. This theoretically derived hypothesis was in fact proposed in [20,21], based on experiments studying diauxic shift. The molecular basis of this observation was recently discussed in [17,18], and our findings support this.

**Fig 1:**
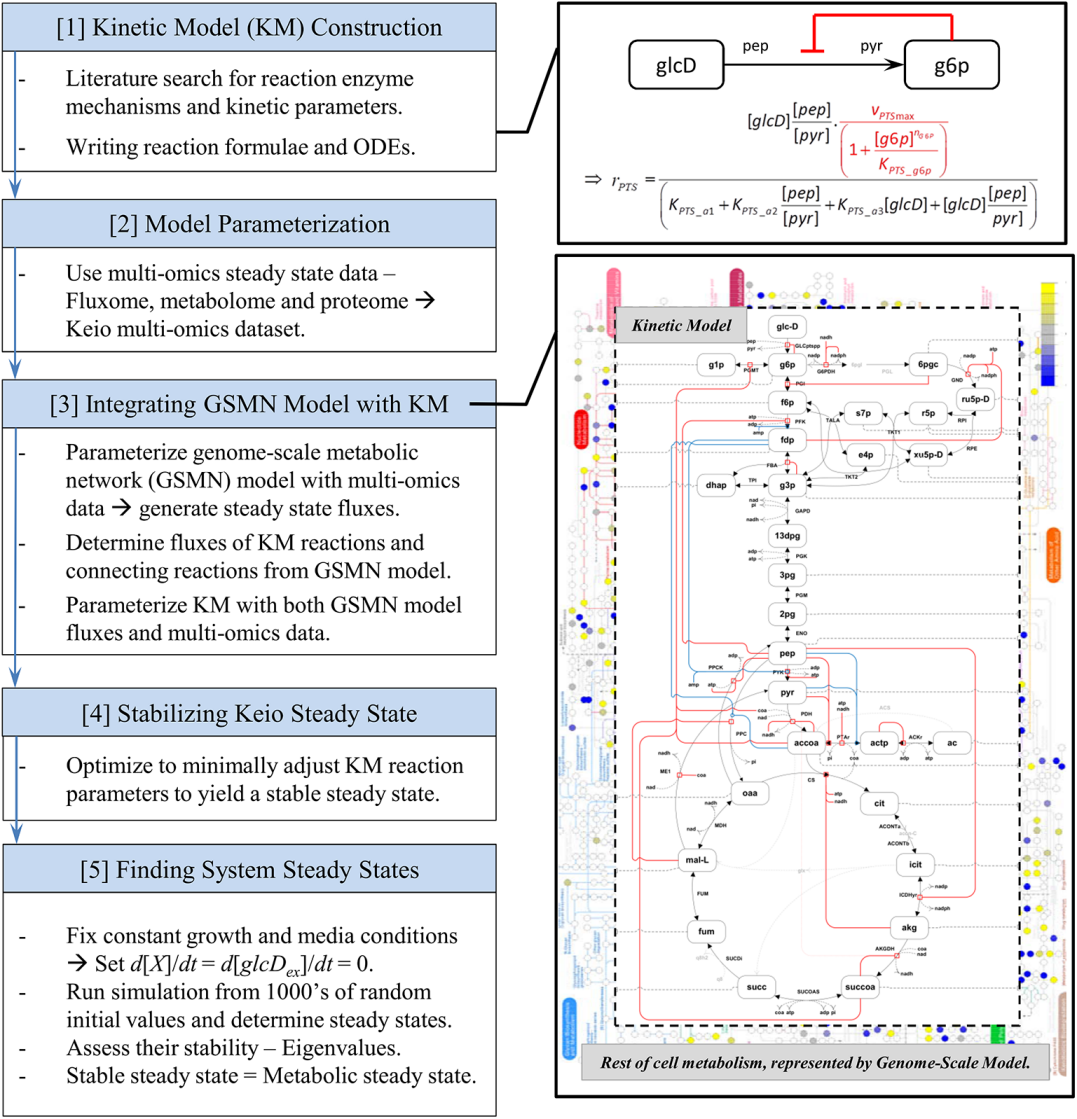
Work flow of kinetic model construction, integration and analyses. A flow diagram of the steps taken and methods implemented for the kinetic model construction, parameterization and integration with the genome-scale model. The diagram also details the techniques utilized for the mathematical analyses of the model for unveiling its systemic properties. Further details are presented in the Materials and Methods section.

In the following we present first our novel integrated kinetic model. We construct the model by integrating the kinetics with the rest of metabolism and growth, and demonstrate how this integration is achieved through the acquisition of physiologically meaningful steady state fluxes from a parameterized GSMN model. Secondly, we demonstrate the power of our integrated kinetic model by applying dynamical systems analysis to gain insight into the biological phenomenon of coexisting metabolic phenotypes. A schematic of the outline of our approach is presented in Fig 1.

## Results

Here we present three main outputs from our study: 1, a novel and detailed kinetic model of the central carbon metabolism of *E*. *coli*, for simulating steady state growth conditions; 2, a parameterized form of the GSMN model of *E*. *coli*, which was then used to integrate flux data and parameterize the kinetic model; and 3, the elucidation of the bi-stable nature of central metabolism. This final output demonstrates the application of the model for gaining insight into the emergence of heterogeneous populations at steady state growth conditions.

### The Kinetic Model and Closing the Open System It Represents

A key result of our work is the construction of the most comprehensive and large kinetic model of the central carbon metabolism of *E*. *coli* to date. The model is large in that it represents the whole of central carbon metabolism, as opposed to modelling only a particular pathway such as glycolysis [9,10] or the tricarboxylic acid (TCA) and glyoxylate cycle [13]. Only a couple of models in the literature include reactions from the whole of central carbon metabolism, such as those reported in [8] and [4]. Those models comprise of 30 reactions and 24 metabolites, and 45 reactions and 37 metabolites, respectively. Our kinetic model is comparable in scale, in that it simulates the dynamics of 37 reactions and 30 metabolites from the whole of central metabolism. Like models [4,8] our model includes reactions of the following set of metabolic pathways: glycolysis, the pentose phosphate pathway, the TCA cycle, the glyoxylate shunt and anaplerotic reactions (to enable an account of gluconeogenic flux distribution), and reactions to acetate production. However, unlike the models of [4,8], our kinetic model incorporates all known details of the kinetic mechanism and metabolite regulatory action of each enzyme. The reaction equations therefore account for regulations such as product inhibition, substrate activation, or competitive inhibition to substrate, for instance, as detailed in S2 Table. These details make the model proposed here the most detailed and comprehensive to date.

**Fig 2:**
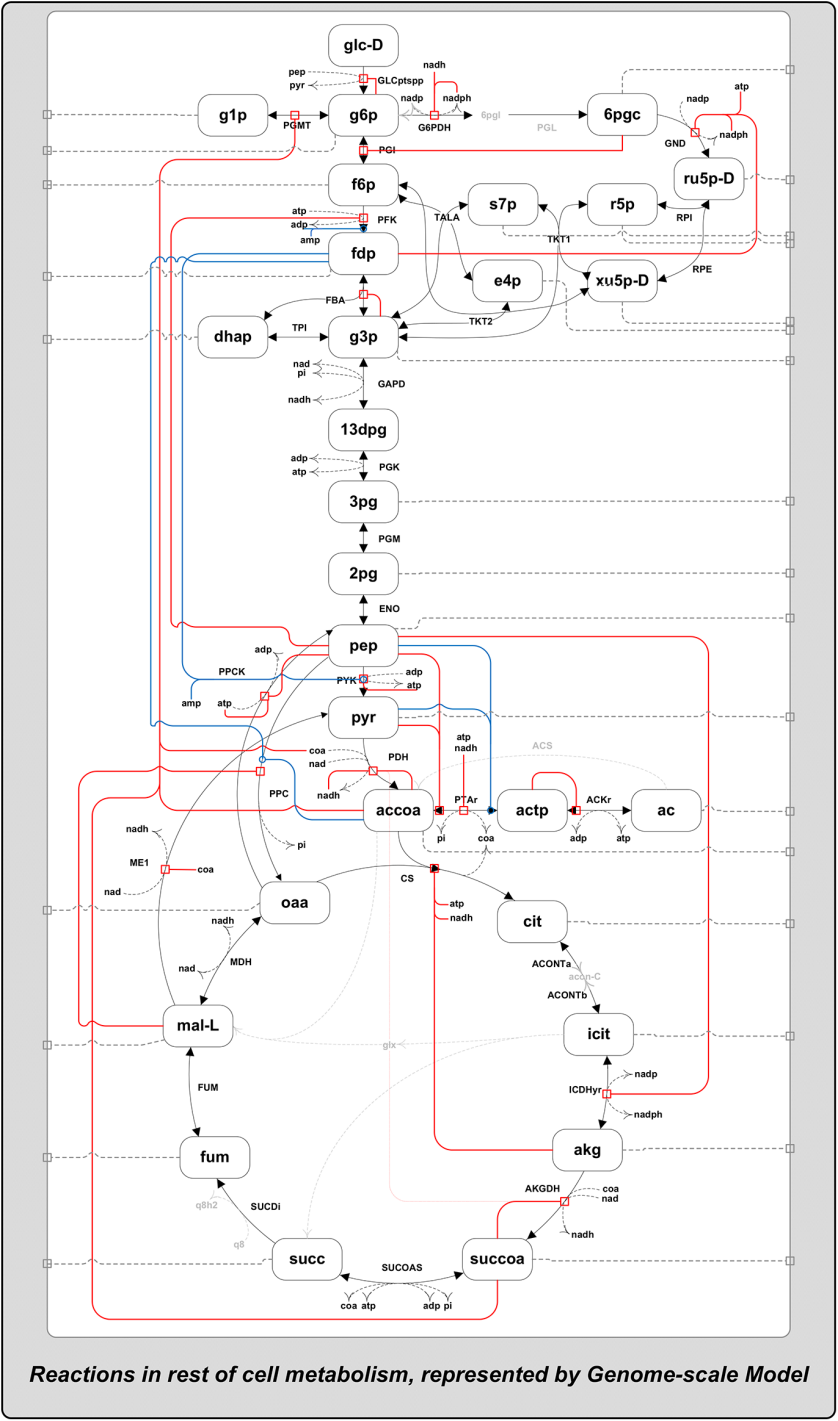
Schematic of the kinetic model reaction network with metabolite regulation. A schematic of the network of reactions and metabolites of the kinetic model, including the metabolite regulation of the respective reactions. The red and blue lines represent enzyme kinetic regulation by metabolite inhibition (red) and non-essential metabolite activation (blue), respectively. Grey dotted lines represent the account of net flux from the reactions connecting metabolites to the rest of the genome-scale metabolic network (connecting reactions).

We adopted a bottom-up approach for the meticulous reaction-by-reaction construction of the kinetic model, as detailed in Materials and Methods. Knowledge of reaction enzyme mechanisms and their regulation were extracted from the literature, and sourced from models [8] and [9] and enzyme databases EcoCyc [22] and BRENDA [23]. This was followed by a subsequent revision of the mathematical description of every reaction, given in S2 and S3 Tables. A schematic of the model, its reactions and their metabolite regulations is shown in Fig 2. Some of the model kinetic parameters were estimated from purified enzyme kinetic data from the literature, where possible, but most were sourced from BRENDA [23]. With multiple values for single kinetic parameters available in BRENDA we selected the parameter values measured from *in-vitro* conditions most closely matching the conditions of our interest. These conditions were those in which the steady state data we use to parameterize the model were measured in. This approach was taken in an attempt to maximize the consistency between the conditions from which all data is derived. As a result, our model represents kinetics under a particular physical condition, namely an aerobic environment, with temperature of 37°C and pH 7.0 [15,16].

Our kinetic model simulates the dynamical response of a single cell to glucose availability in the media, during aerobic steady state growth conditions. In particular the model describes the change of cell biomass production, and the dynamical response of 29 intracellular metabolite concentrations and 37 fluxes of reactions in the central carbon metabolism. The model is formulated mathematically as a coupled system of ordinary differential equations describing the time evolution of cellular biomass [*X*], based on cell specific growth rate *μ*, (Eq 1), the concentration of glucose available in the media (a population level variable) [*glcD*_*ex*_] (Eq 2), and the *i* ^*th*^ intracellular metabolite concentration [*m*_*i*_] (Eq 3):

$$\frac{d[X]}{dt}=\mu \cdot [X]$$ (1)

$$\frac{d[glcD_{ex}]}{dt}=0$$ (2)

$$\begin{array}{l} \frac{d[m_{i}]}{dt}=\left(\sum_{j=1}^{R}s_{ij}\cdot r_{j}\left([\underline{m}];\left\{p_{j}\right\}\right)+c_{i}\right)\cdot \rho_{X}-\mu \cdot \left[m_{i}\right],\;\text{for}\;i=1,\ldots ,M\\ \text{with}\;c_{i}=\sum_{k}s_{ik}\cdot r_{k},\;\text{for}\;k\in \left\{\text{Connecting reactions of metabolite}\;m_{i}\right\} \end{array}$$ (3)

Terms *μ*, *ρ*_*X*_ and [*glcD*_*ex*_] are constants. Eqs 1 and 3 represent the dynamics of the single cell. The units of the variables of the model, describing the dynamical production of biomass and metabolites, are therefore normalized to the volume of the single cell. Eq 2 simply describes the assumed constant availability of external glucose in the media. Eq 3 represents the time evolution of intracellular metabolite concentrations, described by the sum of all reactions weighted by the stoichiometry coefficient of the respective reaction. Each of the reaction equations, *r*_*j*_, are functions describing the reaction enzyme kinetic mechanism, and are dependent on the vector of all metabolite concentrations [*m*], and a set of kinetic parameter values, {*p*_*j*_}. During growth the size of the cell increases for an increase in biomass, which results in an effective dilution of the concentrations of the intracellular metabolites [24]. This effect is accounted for by the final term of Eq 3. Finally, the fixed constant *ρ*_*X*_, representing cell density, is used correct units of metabolite concentration so that concentration are in units of mmol per gram of dry cell weight, per hour. A more detailed explanation of the equations, their respective terms, and units balancing can be found in S1 File. One can of course extend this set of equations to describe the average population level dynamics at steady state growth in the chemostat, as discussed in S3 File.

Further to the high level of kinetic detail incorporated into each reaction equation, the key novelty of our kinetic model is the addition of the term *c*_*i*_, as shown in Eq 3. The kinetic model is a subnetwork of the whole of metabolism, and metabolites whose dynamics it models are consumed and produced by other reactions of metabolism. This contribution from the rest of metabolism is only partially accounted for, at best, by models in the literature, such as that of [9]. As a consequence, we are left with an inherently open system, which also suffers from not incorporating growth rate explicitly. Growth then has to be modelled phenomenologically. The introduction of the fixed constant *c*_*i*_ solves this problem. It enables us to account for the net steady state production of kinetic model metabolite *m*_*i*_, contributed from the rest of metabolism via their respective ‘connecting reactions’ *r*_*k*_−reactions connecting the subnetwork of the kinetic model to the rest of metabolism–as defined in Eq 3.

Our kinetic model will be used to model central carbon metabolism during steady state growth in a media composed initially of glucose as the sole carbon source. As such the pathways of central carbon metabolism included in our kinetic model are the first set of pathways that break down glucose to biosynthetic precursors. These reactions can thus be thought to drive the rest of the cell metabolism. It is currently impossible to model the change in flux of reactions contributing to or consuming metabolites of the kinetic model. This is a common limitation of kinetic modelling. Even in models such as those of [9] and [4] a select few of the metabolites have fluxes phenomenologically representing a drain or contribution to metabolite pools from the rest of metabolism. Most of those fluxes are actually constants while two or three are concentration dependent. Since we are interested in the steady states of central metabolism during fixed constant growth conditions (fixed glucose availability and growth rate), we assume that the contribution to metabolites from the rest of the metabolism is also constant. We thus only need the steady state flux values of the reactions in the whole of metabolism under the constraints of a fixed rate of growth and glucose uptake.

In order to calculate *c*_*i*_, we required steady state flux values of the connecting reactions. These were calculated from a steady state flux balance analysis [25,26] of a genome scale metabolic network (GSMN) model, after fixing the steady state flux values of the central metabolic reactions and growth rate (as detailed in Materials and Methods). The core aspect of any GSMN models is the account of the whole metabolic network topology, where all reactions are effectively connected with each other. One can thus observe how the change in one reaction affects the flux through every other reaction, including that of growth rate. The use of a GSMN model for calculating the connecting reaction flux values therefore addressed three problems simultaneously: 1, it allowed us to account for all known connecting reactions to each kinetic model metabolite, with a total of 271 connecting reactions found from the GSMN model; 2, it enabled a direct association of growth rate to the given flux distribution, as calculated from the model pseudo-reaction representing biomass production rate [27]; and 3, fluxes calculated from it are constrained by network topology and reaction thermodynamics [6]. The thermodynamics of reactions in this GSMN model are based on the knowledge of the reaction Gibb’s free energy and constrain reaction direction. The reaction thermodynamics is therefore accounted for by constraining the reaction flux lower bound to 0 for irreversible reactions and allowing for negative flux value for reversible reactions [6]. The reaction flux upper bound is unconstrained for intracellular reactions. Calculating the fluxes of the connecting reactions in any other way neither guarantees a direct account of growth rate or ensures that thermodynamic and topological constraints of the flux distribution of the whole metabolic network (especially as far as the connecting reactions are concerned) are satisfied.

Since any GSMN model would grant us these advantages over a phenomenological account of the rest of metabolism, to calculate the net flux value of *c*_*i*_ we choose to use the latest and most comprehensive GSMN model of *E*. *coli*, namely the *iAF1260 E*. *coli* GSMN model [6]. To ensure that the fluxes calculated from it simulated our growth conditions of interest (aerobic steady state growth, in a media of 37°C and pH 7.0, with glucose as the sole carbon source) it was critical to first parameterize this model. This is discussed in the next subsection.

### Generating Meaningful Steady State Fluxes from the GSMN Model

Calculation of the metabolic flux values of the connecting reactions in the kinetic model will be obtained from a flux balance analysis of the *iAF1260* GSMN model of *E*. *coli*. These values will then be integrated into the kinetic model via the determination of the parameters *c*_*i*_. To enable this integration two important features had to be accounted for. Firstly, the kinetic model and the GSMN model must represent metabolism for the same steady state growth conditions, preventing a qualitative discrepancy between the flux distributions of the two models. Secondly, steady state fluxes of reactions in the kinetic model must be the same as the same set of reactions in the GSMN model. This will prevent a quantitative discrepancy and incompatibility, and enable the explicit integration of flux values between the two models.

**Fig 3:**
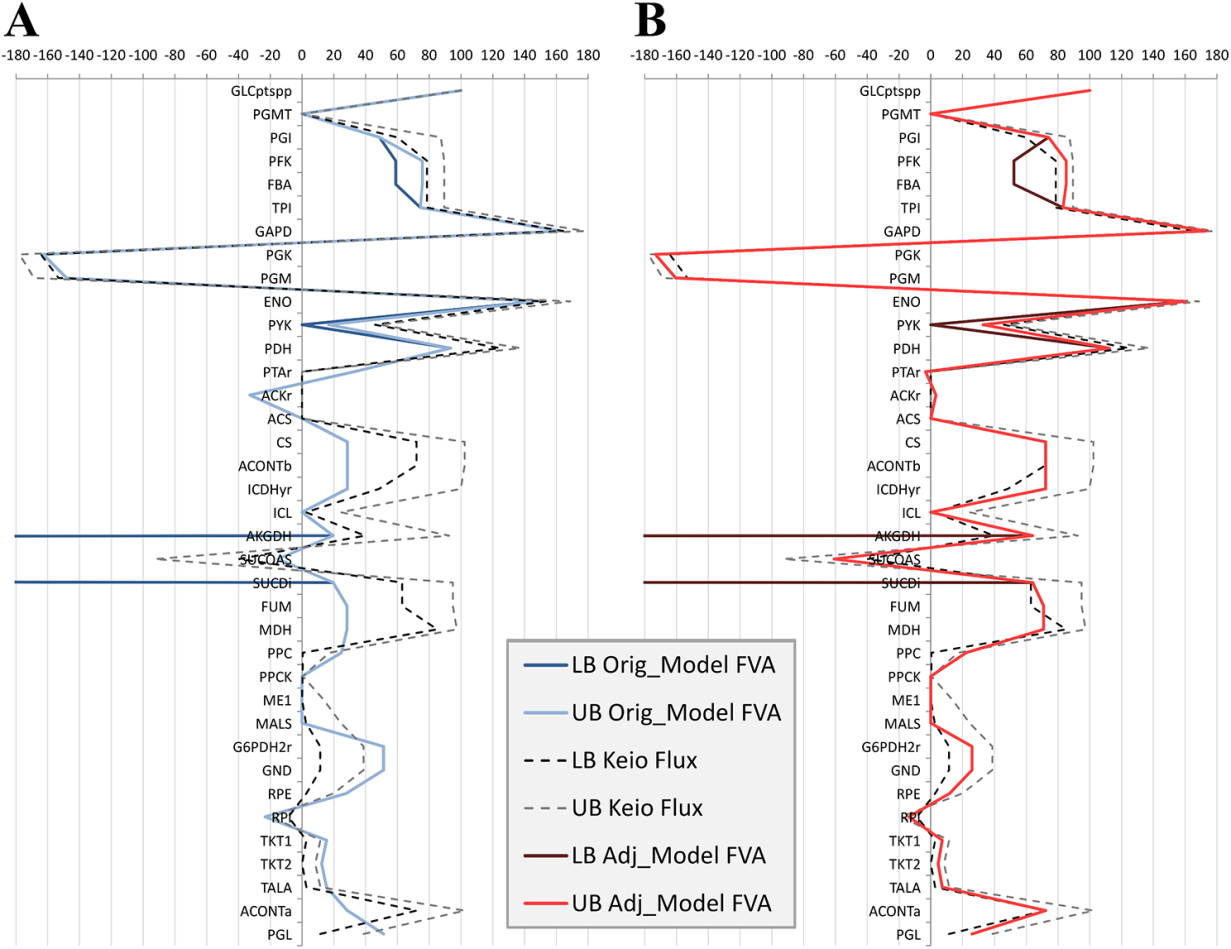
Comparison of relative flux ranges predicted by the model and those reported in Keio database. Both plots [A] and [B] compare upper and lower flux values (relative to GLCptspp reaction) predicted from a flux variability analysis of the genome-scale model (solid lines) and those reported from the carbon-13 metabolic flux analysis in Keio database (dotted lines). Lower flux values are darker lines and upper flux values are lines lighter in shade. The range of flux based on the Keio database was calculated as the variance of 4 replicates; upper (grey dotted line) and lower bound (black dotted line) is +/- 2 standard deviations from the mean, respectively. [A] The comparison is made using the original *iAF1260 E*. *coli* genome-scale model, before reparameterization. [B] The comparison is made using the optimally reparameterized version of the *iAF1260 E*. *coli* genome-scale model.

To ensure that both models represent the same steady state growth conditions and output the same fluxes of reactions in central metabolism we needed to parameterize both models using data taken from the same steady state growth experiment. For this we turned to the multi-omics data from the Keio database [16], for growth rate 0.2 *h* ^*-1*^. In particular we focused on the reported fluxomics data, which were experimentally estimated from carbon-13 metabolic flux analysis [28]. However, determination of these fluxes was not based on the genome scale metabolic network. Therefore, constraining the reactions of central metabolism in the GSMN model to their respective reported values from the fluxomics data resulted in a violation of the topological and thermodynamic constraints of the model. In fact, there was a significant qualitative difference between the fluxes of reactions in the central metabolism reported in the fluxomics data and those determined from a flux balance analysis of the GSMN model, as is apparent from Fig 3A.

To overcome this problem, we adjusted the GSMN model parameters such that an evaluation of the model reproduced qualitatively the experimentally derived fluxomics data. The flux values of reactions in central metabolism determined from a flux balance analysis of the adjusted GSMN model do of course perfectly balance with fluxes of the rest of metabolism. This is precisely what enables the direct calculation of the required connecting reaction flux values, giving us the integration of the steady state flux distribution of the rest of metabolism to the kinetic model. It is precisely the ability to take both these values from the same GSMN model that ensures the thermodynamic consistency between fluxes of the kinetic model and rest of metabolism.

The GSMN model contains two sets of ‘strain specific’ parameters [29]. The first set is composed of reaction flux bounds. This includes the bounds of glucose and oxygen uptake reactions, biomass production reaction (growth rate), and ATPase reaction, representing non-growth associated maintenance cost. The second set is composed of defined stoichiometric coefficients of important reactions. These include stoichiometries determining the P:O ratio in the oxidative phosphorylation reactions, and those of the biomass reaction accounting for both biomass composition and the growth associated maintenance costs (for polymerization reactions, for instance). As detailed in Materials and Methods, we defined an optimization problem that searched over values of both sets of parameters, for a fixed and defined biomass production reaction flux of 0.2 *h* ^*-1*^. This required only the minimization of the distance between simulated fluxes of the exchange reactions and growth rate to those reported in the Keio database. To ensure that the optimization was not falling into a local minimum, the problem was initiated from 120 different combinations of initial parameter guesses. The unique set of parameters that resulted in the minimum residual value of the objective was chosen and set in the GSMN model. S2 Fig shows the plot of the optimized fitting between the Keio experimental data and simulations from the GSMN model.

We then determined the flux values of the reactions in central metabolism from the now parameterized GSMN model by flux variability analysis. As illustrated in Fig 3B, the qualitative match of the flux distribution between the re-parameterized GSMN model (solid lines) and Keio fluxomics data (dotted lines) was excellent. The power of the predictive ability of the adjusted GSMN model is demonstrated even more so when we observe that almost all the flux values from the model fell within the range of flux estimates reported in the Keio dataset. This range is defined as the 95% confidence interval of repeated measures.

This result enabled us to extract two sets of data: the steady state fluxes of the reactions in the central metabolism and the steady state flux of the connecting reactions, both used to parameterize the kinetic model. Since both these sets of fluxes are calculated based on the topological and thermodynamic constraints of the genome wide metabolic network, they are also perfectly consistent with fluxes of the rest of metabolism and our steady state growth rate of interest.

The parameterisation of the GSMN model by the re-specification of its strain-specific parameters is therefore powerful enough to enable the model to qualitatively match a realistic steady state flux distribution of the cell metabolism, for the given growth and media conditions. We emphasize that this was achieved without relying on the computationally challenging carbon-13 metabolic flux analysis, and without neglecting the high connectivity of the whole cell metabolism.

### Parameterizing the Kinetic Model

The mathematical description of reaction enzyme kinetics in our kinetic model is of a form similar to Michaelis-Menten or mass action kinetic equations. A description of the enzymatic mechanism of each reaction in the model is given in S2 Table, with the respective mathematical descriptions given in S3 Table.

As detailed in Materials and Methods, we designed a novel approach to the parameterization of the kinetic model. Known steady state metabolite concentrations, [*m*]_*k*_, from Keio multi-omics database, and known parameter values, *k* ^*cat*^ and *p*_*k*_, from literature, were set in each reaction equation. Each reaction equation was then set equal to its respective steady state flux value, *f*_*j*_, determined from an evaluation of the adjusted GSMN model. In general, a reaction equation can be written as follows:

$$\begin{array}{l} f_{j}=r_{j}\left([\underline{m}];\left\{p_{j}\right\}\right)=r_{j}\left({\left[\underline{m}\right]}_{k},{\left[\underline{m}\right]}_{u};{\left\{a_{1j}\cdot p_{j}\right\}}_{k},{\left\{p_{j}\right\}}_{u},v_{j}^{\text{max}}\right)\\ v_{j}^{\text{max}}=a_{2}\cdot k^{cat}\cdot \left[e_{j}\right] \end{array}$$ (4)

Parameterization of the kinetic model not only involved the determination of unknown kinetic parameter values *p*_*u*_, but also unknown steady state metabolite concentration values [*m*]_*u*_. Setting known values and determining the unknown values resulted in an over constrained problem with no solution. However, known parameter values can also be allowed to vary to enable enough freedom in the problem to find a feasible solution. We therefore introduced ‘adjustment factors’ *a*_*1*_ and *a*_*2*_ (in Eq 4), and defined the parameterization problem so as to find feasible values of the missing data from within a defined range of values, whilst minimizing the adjustment to the known kinetic parameters. The defined ranges for missing data served to ensure values chosen were physiologically similar to known metabolite concentration values, as well as ensuring they did not violate the thermodynamics (direction) of the reaction. Known kinetic parameter values were only taken from the literature if they were measured in physiological conditions close to those adopted here (pH 7.0 and 37°C). Thus, the choice to minimally adjust parameter values ensures that the adjusted parameters do not stray far from values that are physiologically close to the conditions of interest. Searching for parameters without further knowledge, or even blindly, will most likely determine values far from those physiologically feasible, compromising the relevance of the results from an evaluation of the kinetic model.

### Advantages of the Integrated Kinetic Model over Typical Models of Metabolism

Our kinetic model represents the dynamical response of central carbon metabolism of a single cell during steady state growth conditions. The subsystem modelled is closed at the boundaries by integrating steady state flux values from a GSMN model. The boundary fluxes thus enable a direct account of both the contribution to metabolite pools from the rest of steady state metabolism and the fixed growth rate, overcoming two inherent problems of kinetic modelling. Furthermore, our model accounts for metabolite regulation of reaction enzyme kinetics to a higher degree than other kinetic models in the literature. Thus, exploitation and analysis of our kinetic model would enable a more informative insight into the steady states achievable by central metabolism, as compared to analysis of other kinetic models.

It is important to realize that the quasi-steady state assumption of enzyme production rates, and by extension gene regulation, means that our kinetic model is limited to representing a short time scale view of the full dynamical response of the cell metabolism during steady state growth conditions. A full dynamical response of the cell would involve the interaction of metabolism with transcription factors, acting as metabolite-flux sensors [11], for instance. Our model cannot be used to simulate changes in substrate availability, change in growth conditions, or batch culture growth. It can only be exploited to elucidate and understand cellular metabolic states under fixed media steady state growth conditions.

Flux balance analysis (FBA) of GSMN models are also used to understand cell steady states for a given growth rate, but only in terms of reaction fluxes. Even then, analysis of the model poses an ill-posed problem with infinitely many solutions of the flux distributions [30]. Moreover, though this modelling has many advantages, for our question of interest, the greatest weakness of the model includes its incapability to predict changes to metabolite concentrations. This disables any insight into the effect that the shift in metabolism has on other components of the cell, such as gene regulation. Other enhancements to FBA, such as dynamic FBA (dFBA) [31] and integrated FBA (iFBA) [32], were developed as a means of integrating kinetics into FBA models. However, such models differ to our integrated kinetic model in two major respects. Firstly, the models are constructed for the purpose of understanding flux changes for changes in media substrate/nutrient availability. Conversely, the purpose of our model is to understand whether the intracellular metabolic state of the cell has the potential to shift to another state under the constraints of fixed substrate availability. Secondly, both these models simulate metabolic changes during batch culture growth conditions, where substrate availability is constantly changing growth rate. Conversely, our model is used to understand whether the cell metabolic state has the opportunity to shift during fixed steady state growth. Therefore, though both dFBA and iFBA models are very useful, for our question of interest they are inappropriate.

### Model Application: Bistability of Central Metabolism and Emergence of Population Heterogeneity

We now demonstrate the power of our kinetic model by applying it to understand changes to the metabolic state of central metabolism of the single cell, during steady state growth conditions. We are particularly interested to gaining an insight into the phenomenon of the emergence of phenotypically heterogeneous bacterial populations. We therefore ask the question of whether central metabolism is able to converge onto more than one metabolic state under the constraints of a fixed steady state growth condition. A change to the metabolic state of the single cell will give rise to a change of its phenotypic profile.

During batch culture growth, bacteria preferentially consume glucose, before switching to the alternative consumption of acetate [14]. During the switch a lag phase in population growth is observed. This lag was believed to occur due to the slow response of gene regulation in all cells in the population. However, recent studies of [17–19] have discovered that the lag was instead caused by the subsequent emergence of a slower growing subpopulation of bacteria. This subpopulation was able to continue to grow on the alternative substrate, whilst the majority of the population failed to continue to grow at all. Cells of the surviving subpopulation can thus be thought to be somewhat preconditioned to growing on the alternative substrate.

In light of this recent insight, we hypothesize that the bacterial population was phenotypically heterogeneous during its steady state growth on glucose (exponential growth phase), prior to the substrate switch. As a precondition to the emergence of the two phenotypes observed in [17–19], we propose the following hypothesis,

H_0_: Central metabolism, the driver of the rest of metabolism in glucose media, is able to converge onto two metabolic steady states. Each steady state would be defined by a distinct metabolite concentration profile. Furthermore, to prove cells in each state can coexist, both states should be achievable even under the constraints of the same media and steady state growth conditions. Due to the fast dynamics of the metabolism as compared to the gene regulatory response, these metabolic states would be achieved and only exist on a time scale which is short with respect to the time scales that characterize the full dynamics of the cell. The subsequent interplay between metabolism, protein interactions and gene regulation would move the metabolic state of the cell towards what would become an observable phenotype. Nevertheless, since the larger and longer time scale shift of the cell phenotype would follow from the shift in metabolic state, it is sufficient to observe a change in the metabolic state to claim a change in the cell phenotype.

We then performed a dynamical systems analysis of the model in order to test for hypothesis H_0_. To test for the existence of two subpopulations we needed to look for two metabolic states that metabolism can converge onto, with every cell having the potential to converge onto either state. To ensure that subpopulations emerging from each metabolic state can co-exist in the same media and growth conditions, we require three conditions to be imposed: fixed growth rate, which is already fixed as a constant, *μ* = 0.2 *h* ^*-1*^; constant glucose availability, which is already defined in Eq 2, d[*glcD*_*ex*_]/d*t* = 0; and a fixed cellular biomass, *d*[*X*]/*dt* = 0. This final condition is critical to enable a direct comparison between the concentration profiles of each metabolic state. Fixing biomass amount to a constant value ensures that we are comparing cells of the same size. Moreover, since cell biomass is proportional to its volume, the set of metabolite concentrations defining each metabolic state are therefore compared over the same cell volume. This enables the direct comparison between metabolic states. As detailed in Materials and Methods, we then searched over the metabolic phase space for stable steady states of the kinetic model, firstly solving for *d*[*m*_*i*_]*/dt* = 0, and then determining its stability from eigenvalue analysis. To ensure that our search for steady states of the system was not always around the same local minima, and to enable a search for solutions over a large span of the metabolic phase space, simulations were initiated over 2000 times. Each time a different vector of initial metabolite concentrations was generated by randomly selecting values for each metabolite concentration from a uniform distribution with interval [0,10]. In this way, we found that simulations converged onto the same two distinct steady states.

**Fig 4:**
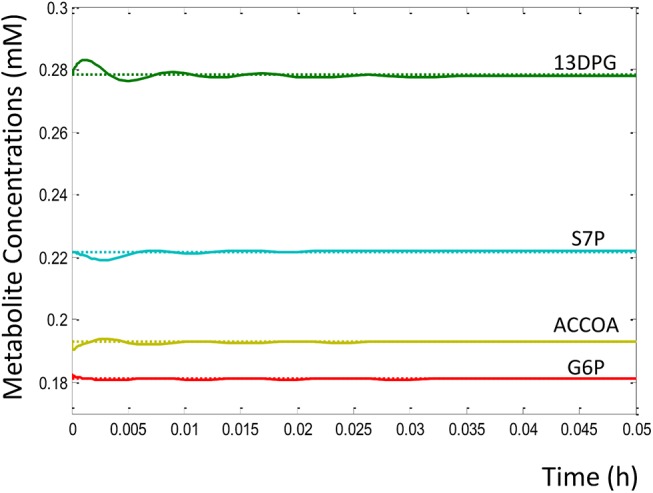
Kinetic model simulation trajectories stabilizing back onto steady state solution. This plot of the trajectories of 4 kinetic model metabolite concentrations over time shows that simulation initiated a small distance away from the steady state allowed the system to relax back onto the Keio steady state (dashed horizontal lines), also proving the state to be stable. Concentrations of all other metabolites also relaxed back onto their respective Keio steady state values, a metabolic profile referred to as the Keio phenotype.

**Fig 5:**
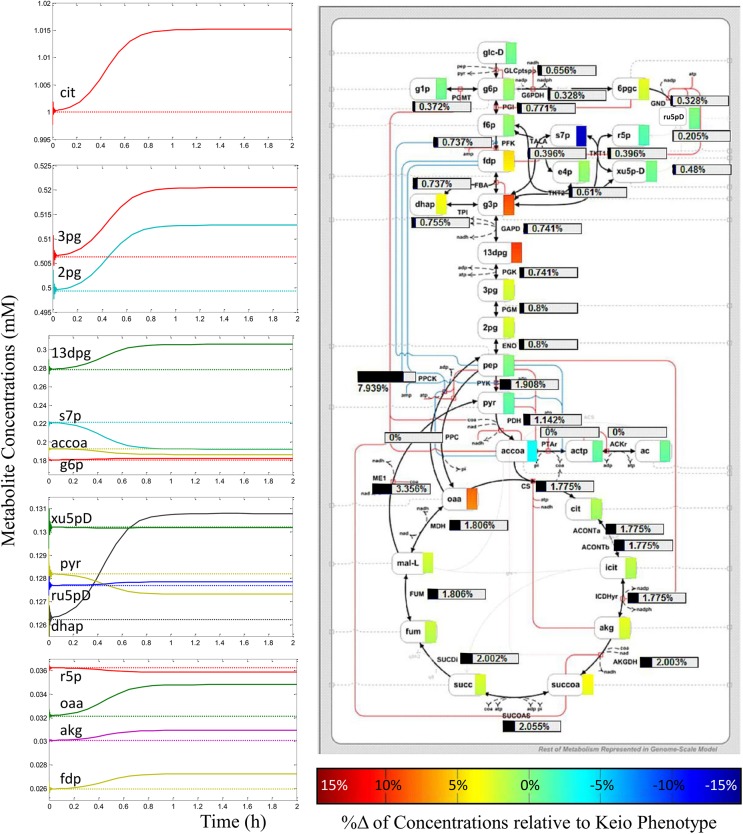
Plots and schematic showing metabolic profile of alternative steady state found. Plots along left illustrate the convergence of the model metabolite concentrations (solid lines) onto a steady state different from the Keio phenotype (dotted lines). Simulations initiated randomly in the metabolic phase space always and only ever converged onto these two stable metabolic steady states. The kinetic model schematic illustrates the percentage difference in metabolite concentrations (coloured rectangles next to metabolite names) and fluxes (black horizontal bars) between the achieved alternative steady state and the Keio phenotype.

Our analysis did in fact discover exactly and only two stable steady states of central metabolism. One of these metabolic states was defined by the metabolic profile of the Keio steady state data. This was expected as these data were used to parameterize the kinetic model. The stability of this ‘Keio steady state’ was confirmed from eigenvalue analysis, where none of the eigenvalues were found to have positive real parts, as shown in Table 1. If any of the eigenvalues had positive real parts, the associated state would be characterised as unstable. An illustration of the stability of this state is shown in Fig 4, where we observe that dynamics initiated close to the Keio steady state values (curves) quickly converged back onto the same state (horizontal lines). A wider exploration of the metabolic phase space saw the kinetic model system converge onto a different metabolic state, discovering an alternative steady state, as shown in Fig 5. Again, eigenvalue analysis reaffirmed the stability of this alternative metabolic state (S1 Table). The distinction in the profiles of the metabolite concentrations and fluxes between the two metabolic states is shown in Table 2 and Fig 5, respectively. One can, in theory, begin to assess parameter dependencies or perform bifurcation analysis to explore the ways in which the system may switch between the two states found. However, this interesting question falls beyond the scope of this paper, and will be addresses in future studies.

**Table 1:** Eigenvalues of the Kinetic Model System at the Keio Steady State (aex = a x 10^x^).

-4.16e-8	-1.92e6	-2.98e5	-2.58e5	-8.35e3–5.58e3i	-7.80e4	-7.03e4
-3.99e4	-3.95e4	-3.77e4	-3.27e4	-2.48e4 + 5.50e3i	-2.48e4–5.50e3i	-8.35e3 + 5.58e3i
-9.69e4	-1.25e4	-1.09e4	-6.58e3	-1.11e4 + 1.64e3i	-1.11e4–1.64e3i	-0.0043 + 54.34i
-290.82	-6.58e3	-1.09e4	-3.64e3	-2.30e2 + 8.00e2i	-2.30e2–8.00e2i	-0.0043–54.34i
0	-2.57e3	-1.71e3	-1.49e3	0		

**Table 2:** Percentage Increase in Steady State Metabolite Concentrations, from the Keio Phenotype to the Alternative Phenotype.

G6P	G1P	F6P	FDP	DHAP	G3P	13DPG
0.964%	2e-8%	0.714%	5.023%	3.610%	9.276%	9.773%
3PG	2PG	PEP	PYR	6PGC	Ru5PD	Xu5PD
2.799%	2.700%	-0.161%	-0.691%	2.943%	0.119%	-0.016%
R5P	S7P	E4P	ACCOA	ACTP	AC	OAA
-1.004%	-13.144%	0.918%	-3.231%	-0.411%	-0.353%	8.297%
CIT	ICIT	AKG	SUCC	SUCCOA	FUM	MAL
1.517%	1.923%	2.912%	2.643%	3.767%	2.165%	2.486%

The central carbon metabolism of *E*. *coli* does indeed possess the ability to express coexisting metabolic steady states, distinct in their metabolic profiles but identical in growth phenotype. This validates our hypothesis and demonstrates that two subpopulations can indeed emerge and exist prior to any substrate shift. Since our kinetic model represents growth at steady state, this means that the resulting emergence of a heterogeneous population occurs during exponential growth phase, in feed-batch growth conditions. We emphasize that this does not imply heterogeneity in populations grown in continuous culture conditions, like those in the chemostat. This is because any change in growth phenotype, observed on the longer time scale of the full cell dynamics, will be diluted out of the chemostat if the growth rate of the phenotype is less than the dilution rate. In such conditions homogeneity of growth phenotype will persist. This is also why we were able to parameterize our kinetic model with the Keio steady state data, which was based on measures taken from *E*. *coli* grown in the chemostat, cultures assumed phenotypically homogeneous.

The striking discovery of exactly two distinct metabolic stable states of central metabolism is in fact the result of the bistable nature of central metabolism. As illustrated in Fig 5, the simulations show the alternative metabolic phenotype clearly expressing an increased flux through the TCA cycle reactions. The most prominent increase was that of the anaplerotic reactions, in the gluconeogenic direction. The main contributing factor to this change seems to be driven by the observed changes at the intersection between glycolysis and the TCA cycle. In particular we observe an increase in the concentration of TCA cycle metabolite oxaloacetate (oaa) and a decreased concentration of acetyl co-enzyme A (accoa). The decreased concentration of accoa creates a bottleneck for flux from glycolysis into the TCA cycle via citrate synthase (CS), thus causing the increased concentration level of oaa. This in turn drives an increased flux in both the anaplerotic reactions, malic enzyme (ME1) and phosphoenolpyruvate carboxykinase (PPCK), in the gluconeogenic direction. This increased flux into glycolysis seems to enhance flux back into the TCA cycle, as evident from the sudden flux increase of the pyruvate kinase (PYK), pyruvate dehydrogenase (PDH) and citrate synthase (CS) reactions, in the glycolytic direction. Though the fluxes of reactions between phosphoenolpyruvate (pep) and citrate (cit) have increased the steady state concentration pool sizes of the intermediary metabolites (pep, pyruvate—pyr, accoa and citrate—cit) have generally decreased. This is of course caused by the increased demand in CS flux due to the increased concentration in oaa, hindering the accumulation of the respective metabolites pools. In summary, analysis of the difference between the two metabolic steady states revealed how the anaplerotic reactions seem to serve as a means of regulating flux from glycolysis into the TCA cycle, regulation acting in a positive feedback fashion. It is precisely this positive feedback mechanism that enables bistability in central metabolism, driving it to converge onto two distinct stable steady states. We thus claim that it is this property of central metabolism that enables the emergence of phenotypic heterogeneity in bacterial populations.

The metabolic state defined by the Keio steady state metabolite concentrations represents an observed phenotype, as the kinetic model was fully parameterized using this multi-omics data. It is important to realize that the alternative metabolic state predicted by the model is, however, not representative of the final state of an observable phenotype. This is because of the assumed time scale difference in the dynamics of metabolism and gene regulation, implicit in the model. We assume that the time scale of the dynamical changes observed in metabolism is much faster than changes at the level of gene regulation. This is why gene regulatory changes are assumed to be at quasi-steady state and enzyme concentrations remain at fixed values. Changes in the profile of the metabolic state of central metabolism will however not remain unchanged when continuing to observe dynamics over a longer time scale. This is because the interplay of the high connectivity between metabolism, protein, and gene regulatory networks of the cell will come into play. This means that the change in metabolic state predicted by our kinetic model is the initial short time scale response of the cell preluding to the full response on much longer time scales. Though on this short time scale both metabolic states have the same growth rate, the role of metabolite flux sensors and their regulation of transcription factors cause a larger scale adjustment that feeds back onto metabolism. This drives a change to the cellular phenotype and its associated growth rate on the longer time scale fully determining the growth of the cell, as demonstrated in the modelling work of [11]. Nevertheless, the ability of metabolism to be able to converge onto an alternative steady state is a required prerequisite to the expression of an alternative cellular phenotype, which is the focus of our argument. As a consequence, the ability of the cell to converge onto an alternative metabolic state will give rise to an alternative phenotype of the cell. Since both metabolic states can be achieved in the same media and growth conditions, and both will eventually drive the cell to different phenotypes, two phenotypically distinct coexisting subpopulations will eventually emerge.

The key enlightening observation made in [17,18] was the continued growth of a bacterial subpopulation, distinct in its phenotypic profile, on alternative substrate acetate. We hypothesize this is because cells of that subpopulation are already consuming acetate, prior to the substrate shift. We suspect that this preconditions those cells to continue growth on the alternative substrate after the depletion of glucose. The behaviour of the cells of such a subpopulation is expected to be defined by at least a slower growth rate, as is well known in the literature [21]. This would mean that if such a subpopulation was to arise its growth would be significantly less than that of a population of glucose consuming cells, making the proportion of acetate consuming cells decreasing over time. The studies presented in [17] have in fact shown that a coexisting subpopulation of bacteria may not even be growing, persisting in a seemingly dormant state until prompted to grow when the substrate it was geared to assimilate becomes available. They called such a growth and survival process a ‘bet-hedging strategy’ [17,18].

Experimental observations from the studies of [20,21] provide evidence to support our hypothesis that a subpopulation of cells is already consuming acetate. In particular a population of isogenic bacteria grown at steady state, in media initially consisting of only glucose as the sole carbon source, showed the emergence of two phenotypically distinct subpopulations. One subpopulation was found to be composed of cells consuming glucose, and the other was found to be composed of cells consuming the acetate produced from the glucose consuming population. The experiments of [21] show this phenomenon even for cultures at low steady state growth rates, namely lower than 0.3 *h* ^*-1*^. To test our hypothesis, we ask whether the alternative metabolic state predicted by our kinetic model would enable the cell to converge on long time scales onto a phenotype associated to growth on acetate.

An understanding of the long time scale response of the cell leading to convergence onto the alternative metabolic state lies in studying metabolite flux sensors. We suggest that metabolite flux sensors drive the continued cellular shift from the alternative state towards a phenotype coexisting with, but distinct to, the Keio phenotype. A number of studies have established the regulatory role of certain metabolites of the central carbon metabolism, whose binding to transcription factors (TF) creates the flux sensors [11,33–35]. In some cases, the TF-metabolite complex either impedes or relieves the inhibiting effect of the transcription factors. Though there may be a number of metabolites that regulate some important TFs, here we will only discuss those affected by the metabolites included in our kinetic model. Three TFs regulated by the two metabolites fructose-1,6-diphosphate (fdp) and pyruvate (pyr) are Cra, PdhR, and IclR, as summarised in Table 3. These TFs play a crucial role in governing the switch of the metabolic flux distribution from the one achieved during glucose consumption to one similar to that achieved during the assimilation of acetate. Furthermore, the accumulating acetate produced may encourage an increased production of acetyl co-enzyme A synthase (Acs), thereby inducing the assimilation of low concentrations of acetate [36].

**Table 3:** 

Transcription Factor (TF) and its Role	Metabolite and its Effect on TF	Reference
**Cra**–Effecting the expression of genes, negatively for genes of glycolytic enzymes, and positively for genes of enzymes in TCA cycle, glyoxylate shunt and gluconeogenesis.	**Fructose-1,6-bisphosphate (fdp)**–Binds to suppress the inhibiting effects of Cra in the expression of genes encoding for glycolytic enzymes.	[34,35,59]
**PdhR**–Supresses the production of pyruvate dehydrogenase complex.	**Pyruvate (pyr)**–Antagonizes the repression effect of PdhR.	[11]
**IclR**–Represses expression of the glyoxylate bypass operon. Transcription of the operon is also induced when *E*. *coli* is grown during acetate accumulation, in exponential phase.	**Pyruvate (pyr)**–Activate the activity of the IclR transcription factor.	[33]

The results presented in Table 2 show that the alternative metabolic state predicted by the kinetic model possesses an increased concentration in fdp and a decreased concentration in pyr. We suggest that this indicates a standoff between a reinforced flux in glycolysis, as induced from the inhibition of Cra by fdp, and a flux distribution resembling the one seen for acetate assimilation. The decreased pyr concentration means a decreased inhibition of TF PdhR. This would eventually result in a reduced flux through the reaction pyruvate dehydrogenase (PDH). Pyr also plays a role in regulating TF IclR. It is important to note that the steady state concentration of pyr observed in our model at the Keio steady state is of the same order of magnitude as that needed for the complete activation of IclR, as reported in [33]. This means that under the Keio state flux through the glyoxylate shunt is maximally inhibited. The relative decrease in pyr, observed when cell state switches to the alternative steady state, would result in a reduced repression of IclR on the ace operon. We therefore hypothesize that the alternative metabolic steady state would induce a shift in metabolism to activate flux through the inactive glyoxylate bypass. This state would move to stabilize itself with the positive feedback from an increased concentration in metabolite glyoxylate, repressing IclR further. The resulting flux distribution, which also enhances activity in the anaplerotic reactions in the gluconeogenic direction, resembles well the flux distribution of *E*. *coli* acetate assimilation, as estimated from the carbon-13 metabolic flux study in [37]. In summary, the shift in metabolic profile from the Keio metabolic state to the alternative metabolic state induces a standoff between the glycolytic and gluconeogenic flux distributions, and so, by extension, a standoff between the consumption of glucose and acetate.

Further to the role of flux sensors, our kinetic model shows two other key pieces of evidence to support our hypothesis that the alternative metabolic state leads to a shift from glucose consumption to one gearing for acetate consumption. Firstly, the increased concentration of metabolite G6P (Table 2) of the alternative steady state indicates an increased inhibition of the uptake of glucose via the phosphotransferase system (GLCptspp reaction). Secondly, the slow accumulation of low concentrations of acetate during growth at the Keio steady state indicates an increasing likelihood of activating enzyme acetyl co-enzyme A synthase (Acs), which would result in the uptake of acetate. The slow accumulation of acetate, maybe even into the media, may sporadically induce the activation of the produced Acs, as reported in [36], and in turn induce the assimilation of acetate. Fast growth of cells from the consumption of glucose would exponentially increase contribution to the pool of acetate, furthering the likelihood of inducing its uptake via ACS reaction. This is precisely the observation made from the experiments reported in [20].

The alternative phenotype towards which the alternative metabolic steady state evolves already seems geared to growth on acetate, whether or not acetate is present. If acetate is not present, we hypothesize that the cell phenotype resulting from the alternative metabolic steady state would be under growth arrest, similar to persister cells, until acetate becomes available. A similar observation was reported in the studies of [17,18]. If acetate becomes present we speculate that this would induce the activation of Acs, thereby ensuring that cells expressing the alternative phenotype actually switch to acetate consumption. Therefore, in the event of the depletion of glucose, only the cells in the alternative state, geared to consuming the alternative carbon source, will continue growth. They will switch immediately and without observation of a ‘lag-phase’, whereas other glucose dependent cells will undergo growth arrest, similar to the observations of [17,18].

In summary, we speculate that the action of the metabolic flux sensors on the alternative metabolic state will cause the cell to converge onto a phenotype that consumes acetate rather than glucose. Ultimately, it is the metabolic flux sensors that will drive the cell to switch from glucose consumption to acetate consumption. This will only follow once the central metabolism is able to shift from the Keio metabolic state to the alternative state. Furthermore the acetate consuming phenotype qualitatively represents those bacterial cells that are hypothesized to coexist with glucose consuming cells prior to substrate shift. Moreover, they were those cells experimentally observed to continue growth on acetate after substrate shift.

## Discussion

In this study we have constructed the most detailed and comprehensive kinetic model of the central carbon metabolism of *E*. *coli* to date. We are aware of only two other models that attempt to model the kinetics of the whole central carbon metabolism by including a number of reactions and metabolites similar to our kinetic model. These models are those reported in [4] and [8]. Like our model they too incorporate reactions of glycolysis, pentose phosphate pathway, TCA cycle, glyoxylate shunt and anaplerotic reactions. The models of [4] and [8] are composed of 45 reactions and 37 metabolites, and 30 metabolites and 24 reactions, respectively, and so are of a size similar to our kinetic model, composed on 37 reactions and 30 metabolites. In addition our model extends the details of the complexity of the interaction between metabolites by also accounting for the details of the enzymatic mechanisms and metabolite regulation of the kinetics of the reactions. It is important to realise the importance and indeed necessity of accounting for enzymatic mechanisms and metabolite regulation of reaction kinetics. Without such an account we would overlook the high complexity of the metabolic interaction network. Emergent properties, such as the ability of central metabolism to express alternative states might never be uncovered. The role metabolism plays in regulating itself then would fall purely in the hands of metabolite-flux sensors, as reported in [11], whereas this may not be the case, as our analysis suggests.

Our construction approach, based on the integration of both steady state multi-omics data and knowledge of reaction fluxes embedded in a parameterized GSMN model, overcame some key inherent problems of kinetic modelling.

Parameterizing the GSMN model with steady state multi-omics data empowered the model to replicate the metabolic flux distribution elucidated from the more involved carbon-13 metabolic flux analysis studies. This ensured that this experimentally informed steady state flux distribution is both thermodynamically consistent with the rest of the cell metabolism, on the genome scale, and is directly associated with our fixed growth rate of interest.

This knowledge was then used to parameterize the kinetic model, closing the inherently open sub-system of metabolism described by the kinetic model. It further ensured that the steady state flux distribution it describes is also thermodynamically consistent with the rest of the cell metabolism. This also means that we achieve a direct account of the growth rate of 0.2 *h* ^*-1*^. Our model construction approach therefore overcame three key inherent problems of kinetic modelling.

Recent observations reported in [17–19] shed light on the mechanisms of how bacterial populations switch between consumption of substrates. In light of these recent insights, the power of the kinetic model was demonstrated to gain an understanding into the emergence of heterogeneous populations during steady state growth conditions. As a precondition to the emergence of population heterogeneity suggested in [17–19], we hypothesized the coexistence of two stable metabolic steady states for a fixed media and growth condition. A dynamical systems analysis of our kinetic model both validated this hypothesis and revealed the bistable nature of central metabolism. The mechanism of this bistability is inherent in the dynamical complexity of central metabolism.

The change in the metabolic profile from the Keio metabolic state to the predicted alternative state occurs on a time scale much shorter than the typical time needed for the cell to shift towards the observable phenotype. On longer time scales, the interplay between gene regulation and metabolism in the cell starts taking place, and is thought to be mediated primarily by metabolite flux sensors. This results in the continued shift of the phenotypic state of the cell on the longer time scale of the full cell dynamics. Based on results from the analysis of our kinetic model, three key effects are relevant here: the decreased concentration of metabolite pyr, the somewhat increased repression of glucose consumption by increased G6P concentration, and the slow accumulation of acetate. The combination of these effects seems to give evidence to the hypothesis that the alternative metabolic state continues to evolve towards a cellular phenotype that switches from the consumption of glucose to that of acetate. Since both the Keio phenotype and the hypothesized alternative phenotype emerge in the same media and growth conditions, they are able to coexist.

The discovery of bistability and its role in the expression of two stable and distinct metabolic steady states under the same conditions raises interesting evolutionary questions: Why has evolution selected for bistability of central metabolism? What advantage could two steady states of the system add to the survival of bacteria?

We conjecture that the alternative steady state indicates a sort of ‘anticipation’ of the need for the cell to transit between the catabolism of glucose and the assimilation of acetate. Intuitively, one may expect that since bacteria have evolved in an environment where the availability of carbon sources are constantly fluctuating, evolution would select for those who are able to make the switch between carbon sources more quickly. Given that the phenotype defined by the Keio steady state faces severe growth attenuation after substrate switching, one may question the role of the Keio steady state. Experimental observation, such as those reported in [21], show that bacteria growing on preferential substrate glucose grow faster than those growing on acetate. This allows the population to grow in size, not only increasing the contribution to acetate production, which in turn increases the propensity for cells to switch phenotype, but this increases the likelihood of the population to contain cells that express alternative phenotypes. This is precisely the ‘bet-hedging’ strategy that evolution has seemingly selected to ensure continuity of the survival of the bacteria.

Intuitively, one would expect that such a bet-hedging strategy plays a more prominent role under nutrient restricted conditions, *i*.*e*. low growth rates. It would increase the likelihood that cells switch phenotypes. In fact, the time required for the system to escape a stable steady state is known to be reduced at lower growth rates, as suggested in [38,39]. We therefore suggest the converse, namely that a decreased propensity for the switch from the Keio phenotype to the discovered alternative phenotype would be observed for conditions giving increased growth rate > 0.3 *h* ^*-1*^.

At higher steady state growth rates, the high glycolytic flux results in an increased steady state pyr concentration, as seen in the Keio database [16]. This results in an increased activation of the *ace* operon inhibitor IclR, strengthening the repression of flux through the glyoxylate shunt. This reduces the ability of the cell to express the gluconeogenic flux distribution. At lower steady state growth rates the concentration of mRNA and protein of Acs is greater [16]. This provides evidence that, though glucose is understood to be the preferred carbon source, this measurement of Acs abundance would suggest a somewhat greater activity of acetate assimilation at low growth rates, as exemplified in our model. This indeed adds weight to our hypothesis that the cell metabolism expresses an increased ability to switching between the consumption of acetate and glucose at low growth rates. To further strengthen this hypothesis, we draw attention to the fact that this phenomenon has in fact been observed experimentally, as reported in [20].

In summary, considering longer time scales of the full dynamics of the cell metabolism, we speculate that the discovered phenotypic heterogeneity serves as a ‘bet-hedging’ strategy to enable the cell metabolism to adjust without growth attenuation. We believe that this supports the phenomenon of the emergence of coexisting phenotypes *a posteriori* to substrate shift, as observed in recent studies [17,18]. The elucidated ability of metabolism to hold a hidden coexisting phenotype, with the purpose of gearing metabolism to adapt more easily to perturbed conditions, is in fact inherent and embedded in the complexity of metabolism itself. This increased complexity in the metabolic reaction network can be seen as a more energy efficient means of implementing the bet-hedging strategy, much like the role of flux sensors [11,35], and so can be envisaged as an evolutionarily derived trait. Here the spared energy may be diverted to conserving growth (preventing growth attenuation on media perturbation), thereby increasing the fitness of the cell.

## Materials and Methods

### Databases, Programmes and Toolboxes Used

For the parameterization of the kinetic model and the reparameterization of the genome-scale model, experimentally measured values and kinetic parameters were extracted and estimated from papers held in the following databases: EcoCyc [22], the BRENDA enzyme database [23], and the *Escherichia coli* multi-omics database [15]. Papers referenced in both EcoCyc and BRENDA were used to elucidate the kinetic mechanism of action; write down or derive the reaction equation, as detailed in S2 and S3 Tables; and extract the respective kinetic parameters, as shown in S4 Table, of a number of reaction equations of the kinetic model.

The parameterization of the kinetic model and reparameterization of the genome-scale model was done using the same set of experimentally measured steady state metabolome, proteome and fluxome data taken from the *E*. *coli* multi-omics database, hereafter referred to as the Keio multi-omics database. This was critical to ensure that both types of models represented the same strain of *E*. *coli* under the same environmental conditions, a necessity for their integration.

MATLAB® R2007b (version 7.5.0) [40] was used as the main platform within which the kinetic and genome-scale models were developed, analysed and evaluated.

To enable the import, manipulation and evaluation of the genome scale model other open-source toolboxes were added to MATLAB. The SBML toolbox [41,42] was used to import and parse SBML and XML files into a MATLAB data structure. Cobra toolbox 2.0 [30] was used to enable standard analysis of the genome-scale models including flux balance analysis, flux variability analysis, and reaction and gene knockouts. The linear programming solver used by the Cobra toolbox functions was that of Gurobi version 4.6.0 [43,44]. Later, as will be further explained, the Gurobi quadratic programming solver was also implemented independent of the Cobra toolbox, via a MATLAB executable function GurobiMEX [45] to solve mixed-integer quadratic programming problems.

For the analysis and evaluation of the kinetic model, the solver ode15s, specific for solving stiff problems, was used from the MATLAB ODE suite [46].

The estimation of unknown steady state metabolic concentrations, unknown kinetic parameters and the adjustment of other known parameters was obtained using the MATLAB optimization toolbox functions fmincon, fminsearch and fminunc [47]. The general form of the objective of the optimization problem was that of minimizing the squared distance between experimental enzyme kinetics data, as extracted from the respective papers for each enzyme of interest, and the curve of the reaction equation. The problem constraints, both equality and inequality, were of a linear form.

### Model Strain and Growth Specifications

The integrated model is parameterized using steady state data from the Keio multi-omics database to ensure that the model is representative of continuous culture growth of the wild-type strain of *Escherichia coli K-12 BW25113* at a dilution rate of 0.2h^*-1*^, under the following environmental conditions: aerobic condition, at a fixed temperature of 37°C and pH of 7.0 [15]; the same conditions under which continuous culture chemostat experiments were performed from which the Keio multi-omics datasets were obtained. 4g/L Glucose was the concentration of the sole limiting carbon source, mixed within a synthetic media of the following specifications: 48mM Na_2_HPO_4_, 22mM KH_2_PO_4_, 10mM NaCl, 45mM (NH_4_)_2_SO_4_, 1mM MgSO_4_, 1mg/L thiamin. HCl, 5.6mg/L CaCl_2_, 8mg/L FeCl_3_, 1mg/L MnCl_2_.4H_2_O, 1.7mg/L ZnCl_2_, 0.43mg/L CuCl_2_·2H_2_O, 0.6mg/L CoCl_2_·2H_2_O and 0.6mg/L Na_2_MoO_4_·2H_2_O [15].

### Kinetic Model Reaction Equations and Initial Parameterization

The form of the kinetic model is as given in Eqs 1, 2 and 3. The precise form of the differential equations of the model is based on the units of the variables of the system, and the related units analysis is shown in S1 File. The term c_i_ is added to the differential equation of each intracellular metabolite to account for the net of flux values of connecting reactions as taken from the steady state flux distribution of the rest of metabolism, which is assumed to be at quasi-steady state. Furthermore, the constant multiplicative factor *ρ*_*x*_ introduces a rescaling to ensure that the units of the left and right hand side of the differential equations are consistent.

The enzymatic mechanism of each of the kinetic model reactions had either been sourced or derived from papers, primarily taken from databases such as BRENDA and EcoCyc, as discussed previously. The mathematical form of each of the reaction equations is given in S3 Table. An overview of the kinetic model construction and parameterization process is illustrated in Fig 1.

Equations of the reactions in glycolysis were mainly taken from [9], whereas the reactions of the pentose phosphate reactions were assumed to be representable by reversible Michaelis-Menten and mass action kinetics. All other reaction mechanisms and their respective equations were either based on published kinetic models or derived from experimental work in the literature, where many of these mechanism were described but no explicit mathematical form of their representation was reported. In such cases, it was found that only a few of the required parameters of the equations were reported, with the remaining estimated from kinetic data reported in the paper from which the respective enzyme mechanism was reported.

An example is that of the irreversible reaction isocitrate dehydrogenase (ICDH): **Icdh**: *Icit*[*c*] + *nadp*[*c*] → *akg*[*c*] + *nadph*[*c*] (+ *co*_*2*_[*c*])

The studies of [48] report that the mechanism of action of the Icdh enzyme obeys a compulsory-order on the binding of the substrates with nadp binding first, acting as an essential activator. Such a mechanism is more generally known as sequential kinetics and in this case would be named as an irreversible bi-bi ordered mechanism. This is similar to the name of the reaction enzyme mechanism given in S2 Table. Under such enzymatic action the affinity of the nadp-enzyme complex for icit is much greater than of the enzyme alone for icit. The reaction equation for this mechanism can be written as follows:

$$v_{Icdh}=\frac{v_{Icdh}^{\text{max}}\cdot \frac{icit}{\alpha \cdot K_{m\_icit}}\cdot \frac{nadp}{K_{m\_nadp}}}{1+\frac{nadp}{K_{m\_nadp}}+\frac{icit}{\alpha \cdot K_{m\_icit}}\cdot \frac{nadp}{K_{m\_nadp}}}$$ (5)

**Fig 6:**
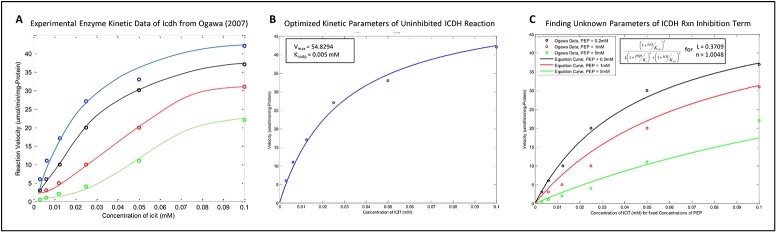
Plots of the optimized parameters determined for ICDH reaction equation. [A] Plot of the experimental measurements of Ogawa *et al*, (2007) (hollow circles), for different fixed concentrations of inhibitor pep ([pep] = 0mM, 0.2mM, 1mM and 5mM are given by blue, black, red and green lines respectively). The solution of the reaction equation with optimized parameters (solid respectively coloured curves) is superimposed onto the data points. The optimization resulted in a very close fit with the measured data. [B] Similar plot, but of only the fitting of the kinetics of the uninhibited reaction. [C] Similar plot, but of only the inhibiting term of the reaction equation, equation given in plot.

As it is found that the affinity of the enzyme for icit is very weak it is thus assumed that no icit-enzyme complex can be formed, hence no term such as *icit/K*_*m_icit*_ is present in the equation denominator. This assumption means that the factor accounting for the increased affinity of the nadp-enzyme complex for icit, *α*, can be assumed to be absorbed into the kinetic value of *K*_*m_icit*_. The kinetic value of *K*_*m_icit*_ = 0.029*mM*, as reported in [49]. The unreported value of *K*_*m_nadp*_ was estimated from the following optimization problem: minimization of squared vertical distance between the curve of Eq 5 and experimental data of the plot of the reaction velocity versus concentration of icit, as reported in [49] and plotted in Fig 6A (blue line). The free variable of the optimization is not only *K*_*m_nadp*_ but is also *v* ^max^_*Icdh*_, since there is no knowledge of how much enzyme was used in the experiments. The free variables are constrained only in a way to ensure that they take positive real values. The optimization was initiated from at least 25 independently generated initial guesses, each value randomly sampled from a uniform distribution. In the case of the *K*_*m_nadp*_ the sampling interval was [0, 10] and for the *v* ^*max*^_*Icdh*_ the interval was [0, 1000]. This was done to ensure that the optimization was not stuck within a single local minima. Nevertheless, each time we found the same unique solution: *K*_*m_nadp*_ = 0.005*mM* and *v* ^max^_*Icdh*_ = 54.829 *μmol/mgProtein/min*. The resulting curve fit is shown in Fig 6B. It was further reported that metabolite phosphoenolpyruvate (pep) played a key role to inhibit the kinetics of this reaction enzyme [49]. To account for the observation that a the shape of the reaction curve becomes more sigmoidal for an increased concentration of inhibitor pep, indicative of allosteric inhibition of icit binding to the nadp-enzyme complex by pep, a Monod-Wyman-Changeux model of cooperativity is assumed, which is adequate since the enzyme is a dimer. To account for the adjustment to the kinetic dynamics a term *R* is multiplied to the Eq 5, where:

$$\begin{array}{l} R=\frac{{\left(1+\beta \right)}^{n}}{L\cdot {\left(1+\gamma \right)}^{n}+{\left(1+\beta \right)}^{n}};\;\beta =\frac{icit}{K_{m\_icit}},\;\gamma =\frac{pep}{K_{i\_pep}}\\ \Rightarrow v_{\;Icdh}=\frac{v_{Icdh}^{\max}\cdot \frac{icit}{\alpha \cdot K_{m\_icit}}\cdot \frac{nadp}{K_{m\_nadp}}}{1+\left(\frac{nadp}{K_{m\_nadp}}\right)+\left(\frac{icit}{\alpha \cdot K_{m\_icit}}\cdot \frac{nadp}{K_{m\_nadp}}\right)}\cdot \frac{{\left(1+\frac{icit}{K_{m\_icit}}\right)}^{n}}{L\cdot {\left(1+\frac{pep}{K_{i\_pep}}\right)}^{n}+{\left(1+\frac{icit}{K_{m\_icit}}\right)}^{n}} \end{array}$$ (6)

This gives us two additional kinetic parameters *L* and *n*. These values were estimated by constructing a similar optimization problem as was done above: minimizing the squared vertical distance between curve of the Eq 6 and experimental data reported in [49], as shown in Fig 6A (black, red, green). We used MatLab's fminsearch function to solve this non-linear unconstrained optimization problem. Three inhibitory kinetic experiments were done in the original paper, each fixing the concentration of pep in the assay at different non-zero concentrations. The objective of the optimization calculates the total of the squared vertical distances between the equation curve and experimental data for each of the inhibitory experiments. We solved the optimization problem initiating from 25 independent guesses of the parameter values, generated from random samples of a uniform distribution of interval size [0, 1000]. We again found that estimates always converged to same unique solution: *L* = 0.3709 and *n* = 1.0048. The curves of the reaction equation with optimized parameters, including those representing the inhibitory effects of pep, are plotted in Fig 6C (solid lines), along with the respective experimental data (dots).

Other reactions for which a similar approach was adopted were: PTAr, PPC, ME1, and the lumped reaction G6PDH and PGL (please see [50] for details). All other reaction equations, along with their kinetic parameters were directly taken or calculated from other published work.

As discussed in [6], 152 reactions of the metabolism of *E*. *coli* correspond to open-reading frames that are understood not to be transcribed under aerobic and glucose limiting growth conditions. 5 of these reactions which are part of the kinetic model were also assumed to be non-active under the given growth conditions: acetyl co-enzyme A synthase (ACS); isocitrate lyase (ICL); malate synthase (MALS); and fumarate reductase reactions, one which depends on cometabolite menaquinol-8 (FRD2) and the other on 2-demethylmenaquinol-8 (FRD3). The expression of genes governing the Acs protein is understood to be mainly observed during cellular growth during utilization of acetate [22,51], which is not the case for our model conditions and thus the activity of Acs is assumed to be zero. The aerobic conditions represented by the model means that both the reactions FRD2 and FRD3, reducing fumarate to succinate, are understood to be repressed [52], and thus their activity is assumed to be zero. The inactivity of these 3 reactions is also in agreement with the flux predictions from carbon-13 metabolic flux analysis results reported in the Keio multi-omics database.

It is understood that the genes governing the expression and production of Icl and Mals enzymes undergo catabolite repression under our conditions of interest [53]. One could thus assume their activities to be inactive, but the reported fluxes of reactions ICL and MALS in the database were however not predicted to be inactive at low growth rates, including the growth rate of our interest, 0.2 h^*-1*^, a prediction also backed by [54]. To test how our assumption of inactive ICL and MALS affects the model flux predictions we opened the upper flux bound of ICL and MALS in the genome-scale model, initially assuming an uninhibited activity through the two reactions (making up the glyoxylate shunt). In the determination of the strain-specific parameters of the genome-scale model, we find that of the range of possible flux values through the glyoxylate shunt reactions, ICL and MALS, are at most 0.005% of the glucose uptake rate. This was done by solving an optimization problem to minimize the distance between experimentally observed uptake rates and simulated uptake rates (the step required before using the steady state fluxes of the genome-scale metabolic network to parameterize our kinetic model, as detailed in the next subsection). This means that our original assumption that there is little or no flux through the glyoxylate shunt reactions, in line with the imposed assumption of [6] and [53], was valid. As such constraining flux through glyoxylate shunt reaction to zero would have an insignificant, if any, impact on the prediction of flux distribution. When comparing the flux distribution between the version of the model with ICL and MALS constrained to zero and the version where the reactions are unconstrained, there was indeed an insignificant absolute difference.

The non-zero flux observed from experiments is based on measuring an average over a sample of bacteria grown in steady state continuous culture. As was emphasized in [21], at low growth rates (below 0.3 h^*-1*^), considered as a state of carbon-source stress, a population of bacteria would contain two subpopulations, one consuming acetate and the other producing acetate and consuming glucose. Though the study of [20] would suggest that a subpopulation of acetate consuming cells is probably always present amongst glucose consuming cells, the proportional abundance of the acetate consuming subpopulation becomes significantly higher at lower growth rates [18]. The subpopulation consuming the available acetate, the pool which is produced and replenished by glucose consuming cells, is understood to have both a different gene-expression profile and non-zero activity through the glyoxylate shunt. Taking measurements averaging over the population would mean that we would observe an activity of the glyoxylate shunt, though it was not present in the subpopulation of cells consuming only glucose. Therefore, it may be that the non-zero flux of the glyoxylate shunt, estimated from chemostat experiments in the studies of [15], was as a result of measurements averaging over a heterogeneous population.

Furthermore the difference in gene-expression profiles between the two subpopulations is also a factor we wish to exclude in our study. We wish to understand whether central metabolism allows the existence of alternative metabolic states at a given steady state condition. By extension we wish to understand whether the cell even has the opportunity to converge onto an alternative phenotype. To gain such an understanding we need to construct our kinetic model to represent one of the phenotypes, namely the steady state of glucose consuming cells. This phenotype will in turn be defined by a fixed gene expression profile specific to glucose consuming cells. We can then analyse the model to understand whether central metabolism has the opportunity to converge onto an alternative state from the glucose consuming state. As discussed in results, we found that indeed the glucose consuming cell can converge onto an alternative state (how does not matter), and that this alternative states is hypothesized to shift phenotype from a cell that consumes glucose to one that consumes acetate.

Modelling glucose consuming cells means that reactions of the glyoxylate shunt are assumed inactive. Two key pieces of evidence enable us to make such an assumption: 1, FBA of the GSMN model predicts insignificant flux through the reactions of the glyoxylate shunt; and 2, the study of [33] indicates that the steady state concentration of pyruvate for glucose consuming cells, taken from the Keio multi-omics dataset, would maximally activate IclR (repressing flux of glyoxylate shunt).We therefore consider the glyoxylate shunt reactions ICL and MALS to be inactive for the purposes of this model. Even though the glyoxylate shunt is inactive, since it is strongly repressed for glucose consuming cells, this does not mean that central metabolism cannot shift to activate it again. In fact, as discussed in the Results section, we find that if a glucose consuming cell converges onto the alternative metabolic state, the decrease in the concentration of pyruvate will lower the activation of IclR and so will release the inhibition of flux through the glyoxylate bypass. We therefore hypothesize that the alternative state will cause the cell to converge onto a phenotype that will have an active flux through glyoxylate reactions, but it is not active yet. This is because that will occur on the longer time scale characterizing the full cell dynamics, a regime in which our model is not valid.

During the construction of the model a number of assumptions are made: 1) the reaction kinetics are valid under specific and fixed media, growth and environmental conditions, as specified in Materials and Methods; 2) there is perfect mixing within both the biological phase (all cellular components and metabolites in the cell cytoplasm and periplasm) and the liquid phase (everything in the liquid medium outside of the cell); 3) the net change in concentration of cofactor metabolites is zero, where the cofactor metabolites included in the model are atp, adp, amp, nad, nadh, nadp, nadph and coenzyme-A (coa); and 4) over the time-scale of the model simulation there is also no change in either the total concentration of enzymes (fixed gene regulation) or enzyme-substrate complexes (quasi-steady state approximation).

### Reparameterization of Genome-scale Model

The steady state flux value of each of the connecting reactions is required in the kinetic model equations, the term *c*_*i*_ in Eq (3). These values were obtained from a feasible flux balance analysis solution of the genome-scale steady state model of *E*. *coli* of [6]. We discuss how we chose a solution of the flux balance analysis in the next subsection. The objective of the programming problem from which the flux balance analysis solution was found was the minimization of total fluxes, given the fixed growth rate of 0.2 h^*-1*^ and given the steady state fluxes of the reactions of the kinetic model. The key advantage of this objective is that it helps to eliminate futile cycles since it finds an efficient pathway from nutrients to biomass production [55].

To ensure that the flux distribution obtained in the genome-scale model represents that of the same bacterial strain and environmental conditions as those that the kinetic model is constructed to represent, 7 'strain-specific parameters' of the model are adjusted [6,29]: the nutrient uptake rates, as specified by the bounds of model exchange reactions; the maximum oxygen and glucose uptake rates; the atp non-growth associated maintenance flux bounds; stoichiometries specifying growth associated maintenance costs in the biomass production reaction; specification of the P:O ratio, by adjusting stoichiometry of reactions defining the electron transport chain; and the biomass composition, by adjusting stoichiometry of the pseudo-reaction representing biomass production, indeed making the model strain-specific.

The composition of the media in which the bacteria grow is known. All nutrients available in the media specified in section 'Model Strain and Growth Specifications' are assumed to be freely available and non-rate limiting, except glucose and oxygen. The maximum uptake flux bound of the exchange reactions of the respective nutrients in the model were set to 1000, whereas the flux bounds of all other nutrients were set to a zero uptake. Other than the difference of bacterial strain, there is a major difference between the specific growth rates in the original genome-scale model, 0.8–0.9 h^*-1*^, and the one we specify, 0.2 h^*-1*^. Both would be expected to yield a difference in the biomass composition, which thus was required to be changed. The course-grained breakdown of experimentally measured biomass composition was reported in the Keio multi-omics dataset, in units of micromoles per gram of dry cell weight (μmol/gDCW). Using the same methodology as [6], stoichiometric coefficients of known biomass components were replaced with values of the composition from the Keio multi-omics dataset, after converting those to the required units of mmol/gDCW. Other stoichiometric coefficients were assumed unchanged. In order to ensure that the reaction was stoichiometrically balanced to yield 1 mmol/gDCW of biomass, the stoichiometric coefficients were renormalized. This was done by recalculating the weight of each biomass component as a molar fraction, then converting to a weight fraction of 1 unit of biomass resulting in their units being in g/gDCW, and finally converting those values into the required units of mmol/gDCW using the respective molecular weights of the components. A snapshot of the spreadsheet of these calculations is shown in S1 Fig.

Ideally, the specification of the maximum uptake rate of glucose and oxygen, given the growth conditions, would come from the exponential phase of batch culture experiments. Since these are unavailable we leave these bounds as free variables to optimize on. Glucose is the sole carbon source in the media. We thus found that constraining glucose uptake rate, via the glucose exchange reaction, during the optimization, resulted in infeasible solutions. To prevent this from occurring and interfering with the optimization of other parameters the maximum uptake flux bound on glucose was fixed to 1000 to make it non-rate limiting. After all other parameters were found from the optimization problem, the bound on the maximum uptake of glucose in its respective exchange reaction was found. We required our genome-scale model to represent steady state growth at a specific growth rate of 0.2 h^*-1*^, same as the conditions from which the kinetic model was parameterized. This is determined by the maximum exchange flux rate of glucose. We determined the maximum uptake flux of the glucose exchange reaction by defining an optimization, where the objective sought to minimize the squared difference between 0.2 and the specific growth rate of the model. The interval of values within which the choice of the glucose uptake flux was constrained was obtained from the calculation of a 95% confidence interval of 3 replicate measured steady state uptake rates, as from the Keio dataset.

Due to the lack of data, the specification of the exact flux of the non-growth associated atp maintenance reaction ATPM (with both upper and lower flux bounds set to the same value) was left as a free variable. Growth associated maintenance is also assumed to be significantly different given the large difference in growth conditions that we impose and those that were used to parameterize the original genome-scale model. Therefore, the stoichiometries of atp in the biomass production reaction, representing growth-associated maintenance were also left as free variables. In the literature, the mechanism of proton translocation of electron transport is not well established and so the exact number of protons carried are unknown [56]. The P:O ratio in *E*. *coli* can even vary with a change in environmental conditions [57]. Therefore alternative stoichiometries have been proposed for the number of protons translocated from the periplasm to the cytoplasm of the oxidative phosphorylation reaction of the electron transport chain [58]. The P:O ratio can be calculated from the H:O ratio and the H:P ratio, and so any adjustment in the stoichiometry of the oxidative phosphorylation reaction yields a change in the H:P ratio and thus the P:O ratio. The core reactions representing oxidative phosphorylation and the electron transport chain in the genome-scale model are ATPS4rpp, CYTBD2pp, CYTBDpp, CYTBO3_4pp, NADH16pp, NADH17pp, and NADH18pp, where the H:P ratio can be calculated from ATPS4rpp: ATPS4rpp: *adp*[*c*] + 4*h*[*p*] + *pi*[*c*] ↔ *atp*[*c*] + 3*h*[*c*] + *h*_*2*_ *o*[*c*]

The stoichiometry of the protons in the periplasm [*p*] and cytoplasm [*c*] are not precisely known and so can be set as free variables to optimize. It is critical to realize that for this reaction to remain atomically balanced the difference in the stoichiometry of *h*[*p*] and *h*[*c*] must remain 1, with one more proton in the periplasm.

In summary, the following are strain specific parameters of the GSMN model: maximum uptake bounds of the exchange reaction for oxygen; the flux bounds of the atp maintenance reaction; the stoichiometric coefficient for atp in the biomass production reaction representing growth associated maintenance; and the stoichiometry of protons of the oxidative phosphorylation reaction, affecting the cellular P:O ratio. These were left as free variables in the non-linear unconstrained optimization problem, defined as follows: minimization of the sum of the squared vertical distances between the experiment data (from the Keio multi-omics dataset) and the adjusted GSMN model flux solutions. The data from both the sources used in the optimization were the steady state uptake rates of glucose, oxygen and the secretion rate of acetate, over various fixed specific growth rates. To ensure that the solution to the problem was not converging to a local optimum, or becoming stuck in a suboptimal solution, the optimization problem was solved over 120 different initial guesses. The solution, comprising of the parameter estimates that yielded the minimum objective value was selected as the overall solution. Finally, the MATLAB fminsearch function was used to determine the maximum uptake flux value of the glucose exchange reaction, given growth rate was fixed to that of our interest, 0.2*h* ^*-1*^.

### Reparameterization of Kinetic Model in Context of Genome-Scale Model

Kinetic parameters of the equations representing the enzyme dynamics are generally of two types: the Michaelis constants *K*_*m*_, and the maximum reaction fluxes *v*_*max*_. It is important to note that the Michaelis constant is a value representative of the affinity for an enzyme to bind to their respective metabolites. This is a physical property of the enzyme independent of the variation of any other cellular component or system variable. However, to enable us to ensure that the value of *K*_*m*_ remains constant during cell dynamics we must assume the following: the media pH level and temperature remain constant, and that the availability of ions in the media are not rate limiting for any of the enzymes of the system. These assumptions are in line with our core assumption of constant environmental conditions. Furthermore, since *K*_*m*_ represents a property of the enzyme, we assume that its determined value from *in-vitro* experiments is representative of its respective value *in-vivo*. We thus take the *K*_*m*_ value from the literature, as discussed above. The same cannot be said of the *v*_*max*_ values since the parameter is explicitly dependent on the concentration of total enzyme concentration *E*_*t*_:

$$v_{\text{max}}=k_{cat}\cdot \left[E_{t}\right]$$ (7)

for specific enzyme turnover rate *k*_*cat*_. Therefore, instead of taking the value of *v*_*max*_ from the literature we calculated it follows:

$$r=v_{\text{max}}\cdot f\left([\underline{m}];\left\{K_{m}\right\}\right)\Rightarrow v_{\text{max}}=\frac{r}{f\left([\underline{m}];\left\{K_{m}\right\}\right)}$$ (8)

using steady state reaction flux value *r* and steady state metabolite concentrations [*m*] from the Keio multi-omics database, after setting the Michaelis constant values {*K*_*m*_}.

Two problems arose in the calculation of the *v*_*max*_ values:

1. Unknown steady state flux values.
2. Missing metabolite concentrations.

We determined the steady state flux values of each reaction in the kinetic model from the parameterized genome-scale metabolic network (GSMN) model, especially given that the flux distribution of central metabolism was qualitatively the same as that estimated experimentally (carbon-13 metabolic flux analysis). A feasible flux distribution from flux balance analysis of the GSMN model alone can be taken. However we would be left with having to choose a random flux distribution from an infinite number of choices. The flux values we require for our kinetic model are those of the reactions of central metabolism, and the net flux of the connecting reactions.

To determine the fluxes of the central metabolism reactions we looked at the carbon-13 fluxomics (C13-MFA) dataset in the Keio multi-omics dataset [16]. We determined which flux solution to take from a flux balance analysis of the GSMN model by setting up an optimization problem that minimized the distance between the C13-MFA data and the flux solution space of the parameterized GSMN model for the reactions of central metabolism only. This was done by setting up the problem as a quadratic programming (QP) problem with the following steps:

1. The growth rate flux bounds of the genome-scale model were fixed to a value of 0.2 h^*-1*^ (our growth rate of interest).
2. The objective of the linear programming problem was changed to minimize the glucose uptake rate, instead of maximizing the growth rate.
3. From flux variability analysis of this redefined problem we chose and set the minimum acetate secretion and oxygen uptake rates. This was done to ensure that we find the minimal pathway and flux distribution towards our required growth rate.
4. The quadratic programming problem was then defined as the minimization of the squared Euclidean distance between the flux solution space of the redefined genome-scale model and the average of the 13C-MFA flux estimates from the Keio multi-omics dataset.
5. In using programming problem solver Gurobi, via its MATLAB executable function GurobiMEX, the general form of the quadratic programming problem was defined in the following form:

$$\text{max}\;f(\underline{x})=\frac{1}{2}\cdot \left({\underline{x}}^{T}\cdot Q\cdot \underline{x}\right)+{\underline{c}}^{T}\cdot \underline{x}$$ (9)

for the matrix *Q* of the coefficients of the quadratic terms, subject to constraints:

- $A\cdot \underline{x}\leq \underline{b}$, the inequality constraints,
- $E\cdot \underline{x}\leq \underline{d}$, the equality constraints.

The flux values of the central metabolism reactions found from this QP problem was unique, in the sense that it was the only flux distribution that minimizes the distance between the C13-MFA fluxes and GSMN model. We then used these values as our steady state flux values for reactions in the kinetic model.

The net flux of connecting reactions contributing to the pool of each metabolite of the kinetic model is another key parameter of the kinetic model. These were determined from a flux balance analysis of the parameterized GSMN model, with the problem defined as follows:

1. Set the growth rate to 0.2 h^*-1*^.
2. Fix the fluxes of the reactions of central metabolism (those in the kinetic model) to the values as determined above (from a minimization between C13-MFA fluxes and the GSMN model).
3. Set the objective of the programming problem to minimize the total sum of all fluxes.
4. Solve the problem to determine the flux values of the whole GSMN model.
5. Calculate the net flux of the connecting reactions for each kinetic model metabolite, giving us our respective *c*_*i*_ values.

Step 3 and 4 in general do not result in a unique flux distribution of the whole metabolic network, since the degrees of freedom of the ill-posed programming problem far exceed the constraints we set. Note that we are not concerned about the uniqueness of the flux distribution of the whole metabolic network. Our focus is on the flux values of only the 271 connecting reactions. The question remains as to whether the net flux values are unique or not. After constraining fluxes as in steps 1 and 2, we performed a flux variability analysis of the fluxes of the connecting reactions and found a maximum flux range of the order of 10^−4^ of any reaction. This indicated that the fluxes of the connecting reactions exhibit such a small range of variability that it did not make a difference as to which exact flux value was taken–they are the same to within the error of 10^−4^.

As seen from Eq 8, the calculation of *v*_*max*_ requires the knowledge of all steady state fluxes and metabolite concentrations. However, many steady state intracellular metabolite concentration values could not be measured or were not reported in the Keio multi-omics dataset, which brings into focus the second problem. Recalling the explicit form of *v*_*max*_, as given in Eq 7, we can calculate the value of this parameter using the knowledge of the measured kinetic value of either the enzyme turnover rate (*k*_*cat*_) or even the enzyme specific activity. The values of these kinetic parameters of the enzyme were found either in the literature or were taken from enzyme databases BRENDA or EcoCyc, where available. It is important to note that the value of the enzyme specific turnover rate (*k*_*cat*_) was preferred to that of the specific activity since it is a directly measured property of the enzyme, similar to the Michaelis constant. This means that it would not be subject to a re-normalization, which would have been dependent upon the amount of substrate or enzyme used to obtain that value, like it is done for specific activity. Such a re-normalization can introduce errors further to those of experimental or measurement error. However, where the specific turnover rate was unreported we had to resort to using the value of the specific activity.

In using the value of either the enzyme turnover rate or the specific activity, the units of the value of *v*_*max*_ must remain as mmol/gDCW/h, the same units as the reaction flux values. The units of the enzyme turnover rate, specific activity and enzyme concentration are as follows:

1. Turnover Rate $\to \frac{1}{s}$
2. Specific Activity $\to \frac{\mu mol}{mg\text{Protein}\cdot \text{min}}$
3. Enzyme Concentration $\to \frac{mg\text{Protein}}{gDCW}$

Clearly, in order to obtain the correct units of *v*_*max*_ from either of the first two above, its value should be calculated with a scaling factor.

Thus, where the value of *v*_*max*_ could not be calculated using Eq 8, its value was calculated in the following two ways using the steady state enzyme concentrations reported in the Keio multi-omics dataset. The units analyses of each formula is shown below the respective equations to show how their respective scaling factors are used to ensure the correct units of *v*_*max*_:

vmax=TurnoverRate⋅EnzymeConcentrationRespectiveWeightOfPolypeptide⋅36001000=(1s⋅3600)⋅(mgProteingDCWkiloDaltons⋅1000)=1h⋅mgProteingDCWDaltons=1h⋅mgProteingDCWmgmmol=mmolgDCW⋅h (10)

vmax=SpecificActivity⋅EnzymeConcentration⋅601000→(μmol⋅11000mgProtein⋅min⋅160)⋅(mgProteingDCW)→mmolmgProtein⋅h⋅mgProteingDCW→mmolgDCW⋅h (11)

### Stabilizing the Keio Steady State

The parameterised kinetic model is expected to exhibit a steady state profile of metabolite concentrations that are the same as the steady state concentration values from the Keio dataset used to parameterise the model. As a simple check, setting the Keio steady state metabolite concentrations in the ODEs of the kinetic model did indeed find that all ODEs equated to zero, i.e. steady state dynamics. This confirmed that the vector of metabolite concentrations is a steady state of the cell metabolism.

Since this metabolic steady state was observed from experiments then it is expected that this state is stable, i.e. small perturbations away from that state will decay in time, and the system will converge back onto the same steady state. Therefore we need to ensure the stability of the state described by the Keio steady state metabolite concentrations, hereafter referred to as the Keio steady state.

An initial evaluation of the eigenvalues of the Jacobian matrix of the kinetic model found 2 eigenvalues with positive real parts, shown in Table 1, indicating that the Keio steady state is in fact unstable for the given set of parameters. As detailed in the Materials and Methods section, a select few (previously unadjusted) parameters were then minimally adjusted until we satisfied the conditions that the real part of all eigenvalues become ≤ 0. S1 Table shows the result of the optimization, clearly indicating the achievement of the stability of the Keio steady state. A further illustration of the stability of the Keio steady state can be seen in Fig 4 where simulations of the kinetic model metabolite concentrations were initiated a bit away from the Keio steady state. This was achieved by arbitrarily setting the initial concentration of pyruvate to 95% of its Keio steady state concentration. We see that the dynamics (solid lines) quickly converge back onto the Keio steady state values (dotted lines), demonstrating that the Keio steady state is indeed an attractor in the phase space of the system, and is thus stable.

### Determination of Missing Steady State Intracellular Metabolite Concentrations

Given all parameter and steady state reaction flux values it was still not possible to algebraically solve for the missing steady state intracellular metabolite concentrations, since more than one equation containing the same missing metabolite concentration gave different values.

Some reaction equations are only dependent on a single or few of the unknown concentration values, uncoupling them from other equations consisting of other unknown concentration values. This allowed the larger problem to be split into smaller ones: problem 1, finding [2pg] from reaction equations of PGM and ENO; problem 2, finding [6pgc] from reaction equations of PGI and GND; problem 3, finding [cit] and [icit] from reaction equations of ACONTb and ICDH; and problem 4, finding [g3p], [13dpg], [nadh], [xu5p_D], [e4p], [glx], [pi], [actp], and [oaa] from the coupled reaction equations of FBA, TPI, GAPDH, TKT1, TKT2, TALA, PGK, G6PDH, SUCOAS, PTAr, ACKr, PDH, CS, AKGDH, and MDH.

As described above, the value of *v*_*max*_ was deduced from turnover rates and specific activities from literature, as opposed to being determined from experimental data. Therefore, since we do not know what the 'true' values of such parameters should be with respect to our condition specification we allow this parameter to vary in order to find a feasible value of the missing concentration. Where freedom in the variation of these parameters alone was insufficient to determine the missing concentration value, variation in the dissociation constants was also allowed.

To solve for the set of missing concentrations and parameters of the system of equations, of each of the four problems specified above, we defined an optimization problem. The missing concentration values ${\bar{x}}_{unknown}$, the multiplicative adjustment factor of *v*_*max*_, *a*_*1*_, and the multiplicative factor of *K*_*m*_, *a*_*2*_, were all set as free variables of the problem. The objective was defined as follows:

$$\begin{array}{l} \text{min}\;Objective=w_{1}\cdot obj_{1}+w_{2}\cdot obj_{2}+w_{3}\cdot obj_{3},\\ obj_{1}=f\left({\left[\underline{x}\right]}_{k},{\left[\underline{x}\right]}_{u}\;;a_{1}\cdot v_{\text{max}},\left\{a_{2}\cdot K_{m}\right\}\right)-r\\ obj_{2}=a_{1}-1\\ obj_{3}=a_{2}-1 \end{array}$$ (12)

for weights *w*_*i*_. For problems 1–3 the value of *w*_*3*_ = 0, as it was sufficient to adjust only the *v*_*max*_ values, and *w*_*2*_ = 1, so as to leave us with only one weight to find. For these problems the weight was set so as to take the minimum required value to order for at least *obj*_*1*_ to converge to zero, since it is critical for this term to go to zero to avoid discrepancy between the right and left hand sides of the respective reaction equations at steady state.

A similar approach was adopted to determine the weights *w*_*1*_, *w*_*2*_ and *w*_*3*_ for problem 4. However the best option in this case was to leave *w*_*1*_ = 1 and *w*_*2*_ = *w*_*3*_ = 0 so as to allow the optimization to satisfy the most critical constraint. In this case, it should be noted that the values of *a*_*1*_ and *a*_*2*_ were not allowed to vary freely, but were constrained within the closed interval [0, 4], which was found to the minimal interval to allow *obj*_*1*_ to converge to zero.

### Finding System Steady States and Stability Analysis

The system steady state is defined to be the value of all variables (vector of metabolite concentrations) that yield a zero rate of change of those variables:

$$\frac{d[X]}{dt}=\frac{d[glcD_{ex}]}{dt}=\frac{d[m_{i}]}{dt}=0$$ (13)

To find the vector of the steady state metabolite concentrations the MatLab function fsolve was used to numerically solve this coupled system of non-linear algebraic equations. The function required an initial guess from which the solver converges onto a local solution, allowing the opportunity to find alternative steady states of the system.

Due to the nature of the dynamics of the system, with an apparent spread of time-scales, the solution obtained from fsolve converged poorly, since substituting the solution back into the system of differential equations of the kinetic model gave right hand side values of the order of 10^*−1*^ as opposed to zero (within a tolerance of 10^*−6*^). As an alternative, the rough position of steady states were first found using the fsolve function. This profile of metabolite concentrations was then used as the initial conditions to the ODE solver ode15s, which solved the system trajectories till steady state was reached.

Determining the stability nature of each of the steady states found was essential in order to classify whether the respective steady state represented the metabolic profile of a bacterial phenotype, i.e. a stable steady state. To classify the nature of the stability of a given steady state, *m*_*0*_, the numerical Jacobian matrix *J* was constructed and evaluated at the given steady state of interest *m*_*0*_:

$$J=f\prime \left({\underline{m}}_{0},p\right)={\partial_{m}f(\underline{m},p)|}_{\underline{m}={\underline{m}}_{0}}={\left[\begin{array}{ccc} \frac{\partial f_{1}}{\partial m_{1}} & \frac{\partial f_{1}}{\partial m_{2}} & \cdots \\ \frac{\partial f_{2}}{\partial m_{1}} & \frac{\partial f_{2}}{\partial m_{2}} & \cdots \\ \vdots & \vdots & \ddots \end{array}\right]|}_{\underline{m}={\underline{m}}_{0}}$$ (14)

for metabolite concentrations *m*_*i*_ and the function of each differential equation *f*_*i*_. The required partial derivatives were constructed from finite difference calculations based on the definition of the partial derivative, where the partial derivative of the *i* ^*th*^ function *f*_*i*_ with respect to the *j* ^*th*^ variable *m*_*j*_ is given by:

$$J_{ij}={\frac{df_{i}}{dm_{j}}|}_{{\underline{m}}_{0}}=\frac{f_{i}\left(m_{0}^{\left(1\right)},\ldots ,m_{0}^{\left(j\right)}+\varepsilon ,\ldots ,m_{0}^{\left(n\right)}\right)-f_{i}\left(m_{0}^{\left(1\right)},\ldots ,m_{0}^{\left(n\right)}\right)}{\varepsilon }$$ (15)

for perturbation parameter ε, which is taken to be of the order of magnitude of 10^*−10*^.

The eigenvalues of the Jacobian for the given steady state of interest were then determined, λ, by solving:

$$\text{det}\left|J-\lambda \cdot I_{n}\right|=0$$ (16)

with *I*_*n*_, the *n x n* identity matrix. The MatLab function ‘eig’ was used to calculate all eigenvalues of the given Jacobian. If the real parts of every eigenvalue of the Jacobian were less than (or equal to) zero, then the steady state was classified as stable, else it was classified as unstable. If there was a case where all eigenvalues had zero real parts but non-zero complex parts, then the steady state was classified as periodic.

The kinetic model was parameterized using the Keio multi-omics dataset, hence a known steady state of the system is the steady state metabolite concentrations as taken from the Keio steady state. On evaluating the stability of this steady state it was found to be unstable as the real part of two of the eigenvalues of the system Jacobian were found to have positive real parts, as shown in Table 1. Since the Keio steady state was observed, it is expected that the steady state is stable, by definition. Hence it was assumed that the kinetic parameters are incorrect for our conditions of interest, as is apparent from S4 Table.

We setup an optimization problem to minimally adjust a select few kinetic parameters which result in the maximum value of the real part of all eigenvalues of the Keio steady state not to exceed zero into the positive domain. The select few parameters were the dissociation constants of the rate equations that were taken directly from literature along with their respective parameters, where none of these are those parameters already adjusted. They were multiplied by a scaling factor constant *A*_*i*_ for the *i* ^*th*^ parameter of interest. Again, since we have no prior knowledge of the extent by which these parameter values should be adjusted, we construct the objective of the optimization problem to minimize the change to these parameter values. Thus, our overall objective of the optimization is as follows:

$$\text{min}\;Objective=\text{max}(\text{Re}(\lambda ))+0.01\cdot \left\|\underline{A}-\underline{1}\right\|$$ (17)

for the vector of the adjustment terms, *A*, subject to the constraint that each adjustment term *A*_*i*_ should take a value within the closed interval [0.5, 2]. Re(λ) means taking the real part of the complex eigenvalue λ. This will ensure that values are not taken too far from 1, which equates to no change. It is important to realize that the objective is not a continuous function because of the first term of the objective function: max Re(λ), hence it is important to choose an appropriate initiating guess.

## Supporting Information

S1 Fig Recalculation of Biomass Reaction Stoichiometry Coefficients. (PDF)

S2 Fig Reparameterization of Genome-Scale Model. (PDF)

S1 File Units Analysis of Model Equations. (PDF)

S2 File Zipped Folder of MatLab Code of Kinetic Model. (ZIP)

S3 File Kinetic Model of Population Dynamics in Chemostat. (PDF)

S1 Table Eigenvalue Analysis of Steady States. (PDF)

S2 Table Table of Reaction Mechanisms. (PDF)

S3 Table Table of Mathematical Reaction Equations. (PDF)

S4 Table Table of Model Reaction Parameters. (PDF)

## References

[1] Kitano H. Systems biology: a brief overview. Science. 2002 3 1;295(5560):1662–4. 11872829 10.1126/science.1069492

[2] Stelling J. Mathematical models in microbial systems biology. Curr Opin Microbiol. 2004 10;7(5):513–8. 15451507 10.1016/j.mib.2004.08.004

[3] Lazebnik Y. Can a biologist fix a radio? or, what I learned while studying apoptosis. Biochem. 2004 12;69(12):1403–6. 15627398 10.1007/s10541-005-0088-1

[4] Usuda Y, Nishio Y, Iwatani S, Van Dien SJ, Imaizumi A, Shimbo K, et al Dynamic modeling of Escherichia coli metabolic and regulatory systems for amino-acid production. J Biotechnol. Elsevier B.V.; 2010 5 3;147(1):17–30. 10.1016/j.jbiotec.2010.02.018 20219606

[5] Beste DJ V, Hooper T, Stewart G, Bonde B, Avignone-Rossa C, Bushell ME, et al GSMN-TB: a web-based genome-scale network model of Mycobacterium tuberculosis metabolism. Genome Biol. 2007 1;8(5):R89 17521419 10.1186/gb-2007-8-5-r89 PMC1929162

[6] Feist AM, Henry CS, Reed JL, Krummenacker M, Joyce AR, Karp PD, et al A genome-scale metabolic reconstruction for Escherichia coli K-12 MG1655 that accounts for 1260 ORFs and thermodynamic information. Mol Syst Biol. 2007 1;3(121):121. 17593909 10.1038/msb4100155 PMC1911197

[7] Mendum TA, Newcombe J, Mannan AA, Kierzek AM, McFadden J. Interrogation of global mutagenesis data with a genome scale model of Neisseria meningitidis to assess gene fitness in vitro and in sera. Genome Biol. 2011;12(12):R127 10.1186/gb-2011-12-12-r127 22208880 PMC3334622

[8] Kadir TAA, Mannan AA, Kierzek AM, McFadden J, Shimizu K. Modeling and simulation of the main metabolism in Escherichia coli and its several single-gene knockout mutants with experimental verification. Microb Cell Fact. 2010;9:88 10.1186/1475-2859-9-88 21092096 PMC2999585

[9] Chassagnole C, Noisommit-Rizzi N, Schmid JW, Mauch K, Reuss M. Dynamic modeling of the central carbon metabolism ofEscherichia coli. Biotechnol Bioeng. 2002 7 5;79(1):53–73. 17590932 10.1002/bit.10288

[10] Hatzimanikatis V, Bailey JE. Studies on glycolysis—I. Multiple steady states in bacterial glycolysis. Chem Eng Sci. 1997 8;52(15):2579–88.

[11] Kotte O, Zaugg JB, Heinemann M. Bacterial adaptation through distributed sensing of metabolic fluxes. Mol Syst Biol. Nature Publishing Group; 2010 1;6(355):355. 10.1038/msb.2010.10 PMC2858440 20212527

[12] Peskov K, Mogilevskaya E, Demin O. Kinetic modelling of central carbon metabolism in Escherichia coli. FEBS J. 2012;279(18):3374–85. 10.1111/j.1742-4658.2012.08719.x 22823407

[13] Singh VK, Ghosh I. Kinetic modeling of tricarboxylic acid cycle and glyoxylate bypass in Mycobacterium tuberculosis, and its application to assessment of drug targets. Theor Biol Med Model. 2006;3(27). 10.1186/1742-4682-3-27 PMC1563452 16887020

[14] Bettenbrock K, Fischer S, Kremling A, Jahreis K, Sauter T, Gilles E-D. A quantitative approach to catabolite repression in Escherichia coli. J Biol Chem. 2006 2 3;281(5):2578–84. 16263707 10.1074/jbc.M508090200

[15] Ishii N, Nakahigashi K, Baba T, Robert M, Soga T, Kanai A, et al Multiple high-throughput analyses monitor the response of E. coli to perturbations. Science. 2007 4 27;316(5824):593–7. 17379776 10.1126/science.1132067

[16] Tomita M. Escherichia coli Multi-omics Database. Institute for Advanced Biosciences, Keio University 2007 Available: http://ecoli.iab.keio.ac.jp/

[17] Kotte O, Volkmer B, Radzikowski JL, Heinemann M. Phenotypic bistability in Escherichia coli’s central carbon metabolism. Mol Syst Biol. 2014 1;10(7):736. 24987115 10.15252/msb.20135022 PMC4299493

[18] Solopova A, van Gestel J, Weissing FJ, Bachmann H, Teusink B, Kok J, et al Bet-hedging during bacterial diauxic shift. Proc Natl Acad Sci U S A. 2014 5 20;111(20):7427–32. 10.1073/pnas.1320063111 24799698 PMC4034238

[19] Van Heerden JH, Wortel MT, Bruggeman FJ, Heijnen JJ, Bollen YJM, Planqué R, et al Lost in transition: start-up of glycolysis yields subpopulations of nongrowing cells. Science. 2014 2 28;343(6174):1245114 10.1126/science.1245114 24436182

[20] Nikolic N, Barner T, Ackermann M. Analysis of fluorescent reporters indicates heterogeneity in glucose uptake and utilization in clonal bacterial populations. BMC Microbiol. 2013 1;13:258 10.1186/1471-2180-13-258 24238347 PMC3840653

[21] O’Beirne D, Hamer G. The utilisation of glucose/acetate mixtures by Escherichia coli W3110 under aerobic growth conditions. Bioprocess Eng. 2000 10 20;23(4):375–80.

[22] Keseler IM, Collado-Vides J, Santos-Zavaleta A, Peralta-Gil M, Gama-Castro S, Muñiz-Rascado L, et al EcoCyc: a comprehensive database of Escherichia coli biology. Nucleic Acids Res. 2011 1;39(Database issue):D583–90. 10.1093/nar/gkq1143 21097882 PMC3013716

[23] Scheer M, Grote A, Chang A, Schomburg I, Munaretto C, Rother M, et al BRENDA, the enzyme information system in 2011. Nucleic Acids Res. 2011 1;39(Database issue):D670–6. 10.1093/nar/gkq1089 21062828 PMC3013686

[24] Neidhardt FC, Ingraham JL, Schaechter M. Physiology of the Bacterial Cell: A Molecular Approach. Sinauer Associates Inc., U.S.; 1990.

[25] Orth JD, Thiele I, Palsson BØ. What is flux balance analysis? Nat Biotechnol. Nature Publishing Group; 2010 3;28(3):245–8. 10.1038/nbt.1614 20212490 PMC3108565

[26] Holzhütter H-G. The principle of flux minimization and its application to estimate stationary fluxes in metabolic networks. Eur J Biochem. 2004 7;271(14):2905–22. 15233787 10.1111/j.1432-1033.2004.04213.x

[27] Feist AM, Palsson BØ. The Biomass Objective Function. Curr Opin Microbiol. 2010;13(3):344–9. 10.1016/j.mib.2010.03.003 20430689 PMC2912156

[28] Wiechert W. 13C Metabolic Flux Analysis. Metab Eng. 2001;3(3):195–206. 11461141 10.1006/mben.2001.0187

[29] Varma A, Palsson BO. Parametric sensitivity of stoichiometric flux balance models applied to wild-type Escherichia coli metabolism. Biotechnol Bioeng. 1995;45(1):69–79. 18623053 10.1002/bit.260450110

[30] Schellenberger J, Que R, Fleming RMT, Thiele I, Orth JD, Feist AM, et al Quantitative prediction of cellular metabolism with constraint-based models: the COBRA Toolbox v2.0. Nat Protoc. 2011 9;6(9):1290–307. 10.1038/nprot.2011.308 21886097 PMC3319681

[31] Mahadevan R, Edwards JS, Doyle FJ. Dynamic flux balance analysis of diauxic growth in Escherichia coli. Biophys J [Internet]. Elsevier; 2002 9 [cited 2014 Jul 22];83(3):1331–40. Available: http://www.pubmedcentral.nih.gov/articlerender.fcgi?artid=1302231&tool=pmcentrez&rendertype=abstract 12202358 10.1016/S0006-3495(02)73903-9 PMC1302231

[32] Xiao N, Chen TJ, Karr JR, Covert MW, Drive C. Integrating Metabolic, Transcriptional Regulatory and Signal Transduction Models in Escherichia coli ODE Model. 2008;(1):1–7. 10.1093/bioinformatics/btn352 PMC6702764 18621757

[33] Lorca GL, Ezersky A, Lunin V V, Walker JR, Altamentova S, Evdokimova E, et al Glyoxylate and pyruvate are antagonistic effectors of the Escherichia coli IclR transcriptional regulator. J Biol Chem. 2007 6 1;282(22):16476–91. 17426033 10.1074/jbc.M610838200

[34] Ramseier TM. Cra and the control of carbon flux via metabolic pathways. Res Microbiol. 1996;147(6–7):489–93. 9084760 10.1016/0923-2508(96)84003-4

[35] Kochanowski K, Volkmer B, Gerosa L, Haverkorn van Rijsewijk BR, Schmidt A, Heinemann M. Functioning of a metabolic flux sensor in Escherichia coli. Proc Natl Acad Sci U S A. 2013 1 15;110(3):1130–5. 10.1073/pnas.1202582110 23277571 PMC3549114

[36] Wolfe AJ. The Acetate Switch. Microbiol Mol Biol Rev. 2005;69(1):12–50. 15755952 10.1128/MMBR.69.1.12-50.2005 PMC1082793

[37] Zhao J, Shimizu K. Metabolic flux analysis of Escherichia coli K12 grown on 13 C- labeled acetate and glucose using GC-MS and powerful flux calculation method. J Biotechnol. 2003;101(2):101–17. 12568740 10.1016/s0168-1656(02)00316-4

[38] Furusawa C, Kaneko K. A generic mechanism for adaptive growth rate regulation. PLoS Comput Biol. 2008 1;4(1):e3 10.1371/journal.pcbi.0040003 18193939 PMC2186362

[39] Rocco A, Kierzek AM, McFadden J. Slow protein fluctuations explain the emergence of growth phenotypes and persistence in clonal bacterial populations. PLoS One. 2013 1;8(1):e54272 10.1371/journal.pone.0054272 23382887 PMC3558523

[40] MathWorks. MATLAB 7.5.0 (R2007b). Natick, Massachusetts: MathWorks; 2007.

[41] Keating S. SBML Toolbox. California Institute of Technology, Pasadena, CA, USA and EBMLEBI, Hinxton, UK; 2011.

[42] Keating SM, Bornstein BJ, Finney A, Hucka M. SBMLToolbox: an SBML toolbox for MATLAB users. Bioinformatics. 2006 5 15;22(10):1275–7. 16574696 10.1093/bioinformatics/btl111

[43] Gurobi Optimization I. Gurobi Optimization. Houston, Texas; 2011.

[44] Gurobi Optimization I. Gurobi Optimization Reference Manual. Houston, Texas; 2011.

[45] Yin W. GurobiMex: A MATLAB Interface for Gurobi. Available: http://convexoptimization.com/wikimization/index.php/gurobi-mex

[46] MathWorks. Product Support: 1510—Differential Equations in MATLAB. Natick, Massachusetts: MathWorks; 2011 Available: http://www.mathworks.co.uk/support/tech-notes/1500/1510.html

[47] Branch MA, Grace A. MatLab Optimization Toolbox User’s Guide. 24 Prime Park Way, Natick, MA 01760–1500: The Math Works Inc.; 2002.

[48] Nimmo HG. Kinetic mechanism of Escherichia coli isocitrate dehydrogenase and its inhibition by glyoxylate and oxaloacetate. Biochem J. 1986 3 1;234(2):317–23. 3521584 10.1042/bj2340317 PMC1146568

[49] Ogawa T, Murakami K, Mori H, Ishii N, Tomita M, Yoshin M. Role of phosphoenolpyruvate in the NADP-isocitrate dehydrogenase and isocitrate lyase reaction in Escherichia coli. J Bacteriol. 2007 3;189(3):1176–8. 17142397 10.1128/JB.01628-06 PMC1797289

[50] Mannan AA. Integration of Kinetic and Whole-Cell Stoichiometric Model for Hybrid Simulations of Bacterial Central Metabolism. University of Surrey, UK; 2012.

[51] El-Mansi M, Cozzone AJ, Shiloach J, Eikmanns BJ. Control of carbon flux through enzymes of central and intermediary metabolism during growth of Escherichia coli on acetate. Curr Opin Microbiol. 2006 4;9(2):173–9. 16530464 10.1016/j.mib.2006.02.002

[52] Ruiz-Herrera J, Garcia LG. Regulation of Succinate Dehydrogenase in Escherichia coli. J Gen Microbiol. 1972;72:29–35. 4341933 10.1099/00221287-72-1-29

[53] Gerstmeir R, Wendisch VF, Schnicke S, Ruan H, Farwick M, Reinscheid D, et al Acetate metabolism and its regulation in Corynebacterium glutamicum. J Biotechnol. 2003 9;104(1–3):99–122. 12948633 10.1016/s0168-1656(03)00167-6

[54] Fischer E, Sauer U. A novel metabolic cycle catalyzes glucose oxidation and anaplerosis in hungry Escherichia coli. J Biol Chem. 2003 11 21;278(47):46446–51. 12963713 10.1074/jbc.M307968200

[55] Poolman MG, Miguet L, Sweetlove LJ, Fell D a. A genome-scale metabolic model of Arabidopsis and some of its properties. Plant Physiol. 2009 12;151(3):1570–81. 10.1104/pp.109.141267 19755544 PMC2773075

[56] Kim BH, Gadd GM. Bacterial Physiology and Metabolism. Cambridge, UK: Cambridge University Press; 2008.

[57] Neidhardt FC, Ingraham JL, Schaechter M. Physiology of the Bacterial Cell: A Molecular Approach. Sunderland: Mass: Sinauer Associates, Inc.; 1990.

[58] Varma A, Palsson BO. Stoichiometric flux balance models quantitatively predict growth and metabolic by-product secretion in wild-type Escherichia coli W3110. Appl Environ Microbiol. 1994 10;60(10):3724–31. 7986045 10.1128/aem.60.10.3724-3731.1994 PMC201879

[59] Saier MH Jr, Ramseier TM. The Catabolite Repressor/Activator (Cra) Protein of Enteric Bacteria. J Bacteriol. 1996;178(12):3411–7. 8655535 10.1128/jb.178.12.3411-3417.1996 PMC178107

