# Supplementary material for: Integrating Kinetic Model of E. coli with Genome Scale Metabolic Fluxes Overcomes Its Open System Problem and Reveals Bistability in Central Metabolism
Source: PLoS One. 2015 Oct 15;10(10):e0139507. doi: 10.1371/journal.pone.0139507 (PMC4607504; doi:10.1371/journal.pone.0139507)
Supplement: S1 Fig — (PDF) [file pone.0139507.s001.pdf]

S1 Fig: Recalculation of Biomass Reaction Stoichiometry Coefficients

Calculating Molar Mass Of Biomass From Adjusted GSM: A copy of the Feist et al (2007) paper supplimentary File 3, Ecoli CORE Biomass Objective Function

These are the stoichiometric coefficients calculated/normalized in such a way as to produce 1 mmol/gDW of biomass.

Macromolecule	Overall wt% (wild type)	Composition (molar fraction)	mmol/gDW (from Keio)	Metabolite	formula	MW (mg/mmol)	mg (if total 1 mmol)	Composition (Weight Fraction)	g/gDW	StoichCoeffs (mmol/gDW)
Protein	0.55	0.112	0.511785	ala-L	C3H7NO2	89.094	7.930335	7.342%	0.042542	0.598516
		0.047	0.215270	arg-L	C6H15N4O2	175.212	7.377180	6.830%	0.039575	0.251751
		0.053	0.242081	asn-L	C4H8N2O3	132.119	6.021771	5.575%	0.032304	0.283106
		0.053	0.242081	asp-L	C4H6NO4	132.095	6.020504	5.574%	0.032297	0.283106
		0.007	0.031627	cys-L	C3H7NO2S	121.16	0.711162	0.658%	0.003815	0.036987
		0.063	0.288199	gln-L	C5H10N2O3	146.146	8.050249	7.453%	0.043185	0.337039
		0.063	0.288199	glu-L	C5H8NO4	146.122	8.048741	7.452%	0.043177	0.337039
		0.102	0.466045	gly	C2H5NO2	75.067	5.796440	5.367%	0.031095	0.545025
		0.019	0.085696	his-L	C6H9N3O2	155.157	2.562086	2.372%	0.013744	0.100219
		0.053	0.240854	ile-L	C6H13NO2	131.175	5.941683	5.501%	0.031874	0.281671
		0.091	0.418445	leu-L	C6H13NO2	131.175	10.322716	9.557%	0.055376	0.489358
		0.054	0.248290	lys-L	C6H15N2O2	147.198	6.992416	6.474%	0.037511	0.290367
		0.023	0.105228	met-L	C5H11NO2S	149.214	3.009709	2.787%	0.016145	0.123061
		0.037	0.167676	phe-L	C9H11NO2	165.192	5.379891	4.981%	0.028860	0.196092
		0.033	0.152042	pro-L	C5H9NO2	115.132	3.219004	2.980%	0.017268	0.177808
		0.044	0.203616	ser-L	C3H7NO3	105.093	3.865298	3.579%	0.020735	0.238122
		0.051	0.235097	thr-L	C4H9NO3	119.12	5.181821	4.798%	0.027798	0.274938
		0.008	0.034770	trp-L	C11H12N2O2	204.229	1.411496	1.307%	0.007572	0.040662
		0.020	0.093267	tyr-L	C9H11NO3	181.191	3.317775	3.072%	0.017798	0.109073
		0.069	0.316823	val-L	C5H11NO2	117.148	6.846957	6.339%	0.036730	0.370514
DNA	0.031	0.246	0.026170	datp	C10H12N5O12P3	487.151	76.803218	24.982%	0.008169	0.026166
		0.254	0.027020	dctp	C9H12N3O13P3	461.109	72.683241	23.642%	0.007731	0.027016
		0.254	0.027020	dgtp	C10H12N5O13P3	503.15	83.361450	27.115%	0.008867	0.027016
		0.246	0.026170	dttp	C10H13N2O14P3	478.136	74.585484	24.261%	0.007933	0.026166
RNA	0.205	0.240	0.064000	ctp	C9H12N3O14P3	479.124	72.910861	22.579%	0.048772	0.160340
		0.326	0.087000	gtp	C10H12N5O14P3	519.149	112.155056	34.733%	0.075023	0.217963
		0.206	0.055000	utp	C9H11N2O15P3	480.108	62.860468	19.467%	0.042049	0.137792
		0.228	0.061000	atp**	C10H12N5O13P3	503.15	74.982251	23.221%	0.050157	0.152824
murein	0.025	1	0.013890	murein5px4p	C77H117N15O40	1892.848	1892.848000	100.000%	0.026300	0.013894
LPS	0.034	1	0.019450	kdo2lipid4	C84H148N2O37P2	1840.033	1840.033000	100.000%	0.035800	0.019456
lipid	0.091	0.4590	0.022330	pe160	C37H74N1O8P1	691.972	317.610170	46.044%	0.044157	0.063813
		0.5410	0.026320	pe161	C37H70N1O8P1	687.94	372.180489	53.956%	0.051743	0.075215
inorganic ions	0.01	0.7256	0.177600	k	K	38.9637	28.272171	65.884%	0.006918	0.177545
		0.0484	0.011843	nh4	H4N	18.039	0.872831	2.034%	0.000214	0.011839
		0.0323	0.007895	mg2	Mg	23.985	0.773656	1.803%	0.000189	0.007893
		0.0194	0.004737	ca2	Ca	39.9626	0.773416	1.802%	0.000189	0.004736
		0.0290	0.007106	fe2	Fe	55.9349	1.623918	3.784%	0.000397	0.007104
		0.0290	0.007106	fe3	Fe	55.9349	1.623918	3.784%	0.000397	0.007104
		0.0129	0.003158	cu2	Cu	63.546	0.819891	1.911%	0.000201	0.003157
		0.0129	0.003158	mn2	Mn	54.938	0.708828	1.652%	0.000173	0.003157
		0.0129	0.003158	mobd	MoO4	159.94	2.063599	4.809%	0.000505	0.003157
		0.0129	0.003158	cobalt2	Co	58.9332	0.760376	1.772%	0.000186	0.003157
		0.0129	0.003158	zn2	Zn	63.9291	0.824834	1.922%	0.000202	0.003157
		0.0194	0.004737	cl	Cl	34.9689	0.676770	1.577%	0.000166	0.004736
		0.0161	0.003948	so4	O4S	96.062	1.549476	3.611%	0.000379	0.003947
		0.0163	0.004000	pi**	HO4P	95.978	1.568511	3.655%	0.000384	0.003999
soluble pool	0.029	0.1074	0.000576	coa	C21H32N7O16P3S	763.508	82.018017	12.851%	0.000437	0.000572
		0.3415	0.001831	nad	C21H26N7O14P2	662.422	226.201918	35.443%	0.001205	0.001819
		0.0834	0.000447	nadp	C21H25N7O17P3	740.385	61.721763	9.671%	0.000329	0.000444
		0.0416	0.000223	fad	C27H31N9O15P2	783.541	32.586655	5.106%	0.000174	0.000222
		0.0416	0.000223	thf	C19H21N7O6	443.42	18.441376	2.890%	0.000098	0.000222
		0.0416	0.000223	mlthf	C20H21N7O6	455.431	18.940901	2.968%	0.000101	0.000222
		0.0416	0.000223	10fthf	C20H21N7O7	471.43	19.606283	3.072%	0.000104	0.000222
		0.0416	0.000223	thmpp	C12H16N4O7P2S	422.295	17.562810	2.752%	0.000094	0.000222
		0.0416	0.000223	pydx5p	C8H8NO6P	245.127	10.194577	1.597%	0.000054	0.000222
		0.0416	0.000223	pheme	C34H30FeN4O4	614.5729	25.559447	4.005%	0.000136	0.000222
		0.0416	0.000223	sheme	C42H36FeN4O16	1355.912	56.390969	8.836%	0.000300	0.000222
		0.0103	0.000055	udcpdp	C55H89O7P2	924.258	9.480453	1.485%	0.000051	0.000055
		0.0416	0.000223	amet	C15H23N6O5S	399.452	16.612793	2.603%	0.000089	0.000222
		0.0416	0.000223	2ohph	C46H70O2	655.064	27.243430	4.269%	0.000145	0.000222
		0.0416	0.000223	ribflv	C17H20N4O6	376.369	15.652795	2.453%	0.000083	0.000222
		1	93.700200	atp**	C10H12N5O13P3	503.15				93.608376
		1	93.700200	h2o**	H2O	18.015				93.608376
			93.761200	atp**						93.761200
			88.352200	h2o**						84.838026
		1	93.700200	adp	C10H12N5O10P2	424.179				93.608376
		1	93.700200	h	H	1.008				93.608376
		1	93.700200	pi**	HO4P	95.978				93.608376
			0.106365	ppi**	HO7P2	174.949				0.106365
			0.667538	ppi**	HO7P2	174.949				0.667538
			5.348000	h2o**	H2O	18.015				8.770350
			0.773900	ppi**	HO4P	95.978				0.773903
			93.696200	pi**	HO4P	95.978				93.604377

A snapshot of the excel spreadsheet used to recalculate the biomass reaction stoichiometric coefficients, based on the spreadsheet and calculations published by Feist *et al,* (2007). The calculations can be obtained from the respective excel sheet of the supplementary information of the paper of Feist *et al,* (2007).
