# Supplementary material for: Integrating Kinetic Model of E. coli with Genome Scale Metabolic Fluxes Overcomes Its Open System Problem and Reveals Bistability in Central Metabolism
Source: PLoS One. 2015 Oct 15;10(10):e0139507. doi: 10.1371/journal.pone.0139507 (PMC4607504; doi:10.1371/journal.pone.0139507)
Supplement: S2 Fig — (PDF) [file pone.0139507.s002.pdf]

# S2 Fig: Reparameterization of Genome-Scale Model

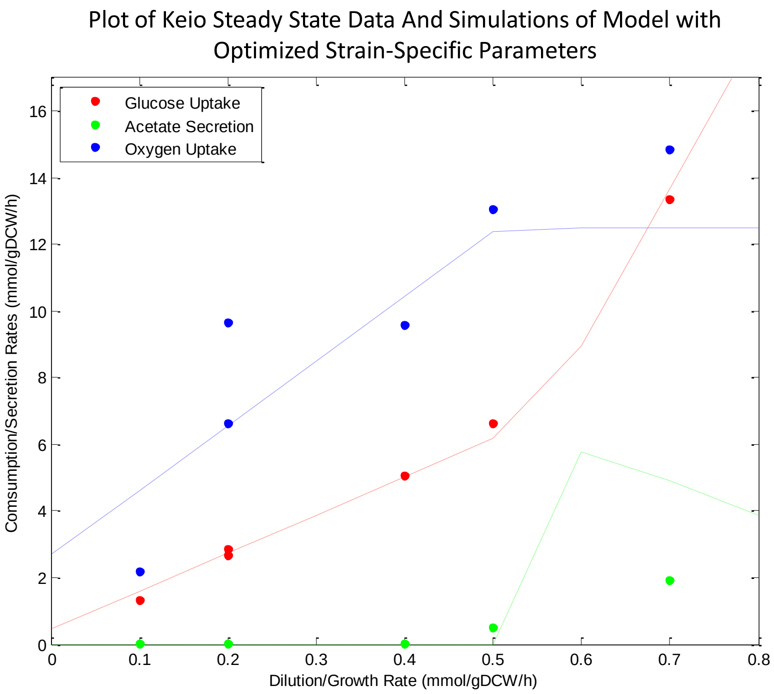


Plot of the optimized fitting between the Keio steady state data (dots) and simulations of the genome-scale model using the optimized strain-specific parameter values.
