# Supplementary material for: Integrating Kinetic Model of E. coli with Genome Scale Metabolic Fluxes Overcomes Its Open System Problem and Reveals Bistability in Central Metabolism
Source: PLoS One. 2015 Oct 15;10(10):e0139507. doi: 10.1371/journal.pone.0139507 (PMC4607504; doi:10.1371/journal.pone.0139507)
Supplement: S1 File — (PDF) [file pone.0139507.s003.pdf]

S1 File: Units Analysis of Model Equations

The form of the system of differential equations of the kinetic model is dependent on the units of both the reaction flux values and metabolite concentrations. We intend to parameterize its reaction equations using the following experimentally measured or derived steady state data reported in the Keio multi-omics database, from Ishii *et al,* (2007):

1. Concentrations of intracellular metabolites, given in units of $mM_c \equiv \frac{mmol}{L_{CellVol}} = \frac{mmol}{L_c}$.
2. Concentrations of extracellular substrates, given in units of $mM_v \equiv \frac{mmol}{L_{CultureVol}} = \frac{mmol}{L_v}$.
3. Concentrations of enzymes, given in units of $\frac{mg-Protein}{gDCW}$.
4. Reaction flux estimates/known values, given in units of: $\frac{mmol}{gDCW \cdot h}$.

Units Analysis of the Dynamics of Biomass

The dynamics of cellular growth is expressed as an exponential growth with specific growth rate μ:

$$\frac{dX}{dt} = \mu \cdot X. \tag{1}$$

With the integration of the kinetic and genome-scale model, the value of μ is taken from the genome-scale model biomass production flux. However, since the flux of this reactions represents the flux of production of $1\frac{mmol}{gDCW}$ of biomass, then value of μ is equivalent to that expected of the specific growth rate, where units would be in $\frac{1}{h}$ as shown:

$$\mu \longrightarrow \frac{\frac{\cancel{\text{mmol}}}{\cancel{\text{gDCW}} \cdot h}}{1\frac{\cancel{\text{mmol}}}{\cancel{\text{gDCW}}}} \longrightarrow \frac{1}{h}. \tag{2}$$

The analysis of the units of the differential equation for the dynamics of biomass concentration thus gives:

$$\frac{dX}{dt} \longrightarrow \frac{1}{h} \cdot \frac{gDCW}{L_v}, \tag{3}$$

which is precisely the units that we need to ensure that the units of the concentration of biomass remains $\frac{gDCW}{L_v}$.

Units Analysis of the Dynamics of Glucose Concentration

The dynamics of the concentration of extracellular substrate glucose, $[glcD_{ex}]$, is expressed by the following differential equation:

$$\frac{d[glcD_{ex}]}{dt} = -r_{PTS} \cdot X, \tag{4}$$

where r_{PTS} is the reaction flux of the uptake of glucose, and X i the biomass concentration. Performing analysis of units on this equation we find:

$$\begin{aligned} \frac{d[glcD_{ex}]}{dt} &\longrightarrow \frac{mmol}{\cancel{\text{gDCW}} \cdot h} \cdot \frac{\cancel{\text{gDCW}}}{L_v} \\ &\longrightarrow \frac{mmol}{L_v \cdot h} = \frac{mM_v}{h}. \end{aligned} \tag{5}$$

The units of the extracellular substrate glucose is thus $\frac{mmol}{L_v} = mM_v$, with respect to the culture volume, not cellular volume, as required. These units are consistent with the units from experimental data measurements.

It is expected that each of the reaction equations are in the expected units of $\frac{mmol}{gDCW \cdot h}$. As an example, we verify that this is indeed the case when performing the units analysis of the reaction equation of r_{PTS},

as taken from Chassagnole *et al,* (2002).

The reaction equation of r_{PTS} is given as:

$$r_{PTS} = \frac{v_{max} \cdot glcD_{ex} \cdot \frac{pep}{pyr}}{\left(\frac{K_A \cdot K_B}{K_C} + K_A \cdot \frac{pep}{pyr} + \frac{K_B}{K_C} \cdot glcD_{ex} + glcD_{ex} \cdot \frac{pep}{pyr}\right)} \cdot \frac{1}{\left(1 + \frac{g6p^n}{K_{g6p}}\right)}. \tag{6}$$

We now analyse the units of this equation (6):

$$\begin{aligned} r_{PTS} \quad &\longrightarrow \quad \frac{\frac{mmol}{gDCW \cdot h} \cdot mM_v \cdot \frac{mM_c}{mM_c}}{\frac{mM_v \cdot mM_c}{mM_c} + mM_v \cdot \frac{mM_c}{mM_c} + \frac{mM_c}{mM_c} \cdot mM_v + mM_v \cdot \frac{mM_c}{mM_c}} \cdot \frac{1}{\left(1 + \frac{mM_c}{mM_c}\right)} \\ &\longrightarrow \quad \frac{\frac{mmol}{gDCW \cdot h} \cdot mM_v}{mM_v} \\ &\longrightarrow \quad \frac{mmol}{gDCW \cdot h}. \end{aligned} \tag{7}$$

Why it is that the units of K_A are in mM_v and not in mM_c like K_B and K_C? K_A is a dissociation constant that represents the affinity between extracellular glucose and its transporter enzyme. Since the transporter is on the membrane and interacts with the extracellular glucose, the affinity it has for the glucose depends on the concentration of the glucose, which in fact is in units of mM_v. The units of K_A must therefore be in the same units.

We have shown that the reaction equation units are indeed in the units that we expect.

Units Analysis of the Dynamics of Intracellular Metabolite Concentrations

To be able to write down the ordinary differential equations to model the kinetics of the 'biological phase'[1] of the cell metabolism a few key assumptions are made:

1. Cell cultures and substrates in the 'liquid phase'[2] are assumed to be ideally mixed.
2. When modelling cell populations, it is assumed that populations are homogeneous. It is hence adequately characterized by an average behaviour, the behaviour predicted by the model.
3. The volume and mass of every cells is assumed constant and equal. Thus, we can lump the interiors of all cells in liquid phase into a pseudo-single cell with an ideally mixed biological phase (Wang *et al,* (2001)).

The dynamics of the intracellular metabolite concentrations m_i, can thus be expressed by the following equation form:

$$\frac{dm_i}{dt} = \sum_j s_{ij} \cdot r_j \cdot \rho_x - \mu \cdot m_i, \tag{8}$$

for reaction equations r_j (similar to r_{PTS}), respective stoichiometric coefficients s_{ij}, and assumed constant cell density ρ_x. The second term of equation (8), $-\mu \cdot m_i$, represents a dilution effect term: concentration of intracellular metabolites is in units of $\frac{mmol}{L_{CellVol}}$. So, as the pseudo-single cell grows ($\mu > 0$ and $[X]$ increases) the concentration of intracellular metabolites effectively become diluted in a greater cell volume.

The constant cell density term ρ_x is calculated from known averaged cellular measurements of wild type *E.coli* cells (Neidhardt *et al,* (1990)):

$$\rho_x = \frac{\text{Single cell dry weight}}{\text{Single cell volume}} = \frac{2.8 \times 10^{-13} gDCW}{4.96 \times 10^{-16} L_c} \approx 410 \frac{gDCW}{L_c}. \tag{9}$$

[1]The Biological Phase: This relates all cellular components and metabolites inside of the cell

[2]The Liquid Phase: This relates to everything in the liquid media outside of the cell.

Given the units of ρ_x and that the units of intracellular metabolite concentrations are $\frac{mmol}{L_{CellVol}} = mM_c$, the units analysis of equation (8) gives us:

$$\frac{dm_i}{dt} \longrightarrow \frac{mmol}{gDCW \cdot h} \cdot \frac{gDCW}{L_c} - \frac{1}{h} \cdot \frac{mmol}{L_c}. \tag{10}$$

It is clear from equation (10) that without the <u>scaling factor</u> of ρ_x the units of the first term would not have been consistent with the units of the second, resulting in a calculation of intracellular metabolite concentration with incorrect units. This demonstrates that indeed the form of the ODEs of the system is required to be appropriately adjusted, with respect to the units of the variables considered, as done here.
