# Supplementary material for: Integrating Kinetic Model of E. coli with Genome Scale Metabolic Fluxes Overcomes Its Open System Problem and Reveals Bistability in Central Metabolism
Source: PLoS One. 2015 Oct 15;10(10):e0139507. doi: 10.1371/journal.pone.0139507 (PMC4607504; doi:10.1371/journal.pone.0139507)
Supplement: S2 File — (ZIP) [file pone.0139507.s004.zip › S2File/README2.pdf]

S2 File: Zipped Folder of MatLab Code of Kinetic Model

The paper is supplied with a zipped folder containing m-files, MatLab readable scripts, containing scripts of the kinetic model itself, its parameters, and code for its analysis. This is aimed at aiding the reader to reproduce the results and figures reported in the results section of the paper. All files are reasonably well commented, with titles to parts of the calculations to explain and make plain to the reader what the functions are doing.

The m-files contained in this folder are as follows:

1. 'InitConds.m':
   The initial conditions set for the kinetic model. These are also referred to as the Keio steady state metabolite concentrations, or Keio Phenotype (since it is a stable state), since they are mostly based on measured metabolite concentrations reported in the Keio multi-omics database from Ishii et al, 2007.
2. 'EColi_Parameters_StableKM.m':
   Parameters used by the kinetic model.
3. 'EColi_StableKM_ContCult.m':
   This is the kinetic model, after ensuring that the parameters of the model give us a stable Keio steady state, as described in the paper. This script contains all the reactions equations and the system of ODEs.
4. 'EColi_ContCult_NoGlcOrXDyn.m':
   This is the kinetic model, same as (3), except that the dynamics of biomass and extraceullar glucose have been fixed to zero. This was done to ensure that we are able to find steady states in the same environmental and media conditions as that of the Keio steady state.
5. 'SystemJacobian.m':
   Function for the calculation of the discrete form of the Jacobian of the kinetic model system, evaluated at a steady state. This matrix is then used to calculate the eigenvalues of the system at the steady state of interest.
6. 'KineticModelAnalysis_ReproduceResultsInPaper.m':
   This is the 'Master Code'. This code is self-contained and contains all functions and calculations that are used to reproduce the results and plots reported in the results section of the paper.
