# Supplementary material for: Integrating Kinetic Model of E. coli with Genome Scale Metabolic Fluxes Overcomes Its Open System Problem and Reveals Bistability in Central Metabolism
Source: PLoS One. 2015 Oct 15;10(10):e0139507. doi: 10.1371/journal.pone.0139507 (PMC4607504; doi:10.1371/journal.pone.0139507)
Supplement: S3 File — (PDF) [file pone.0139507.s005.pdf]

S3 File: Kinetic Model of Population Dynamics in Chemostat

We defined the mathematical form of the kinetic model describing dynamical changes in metabolism and biomass production, for the single cell. The set of equations is given in the paper Results section.

We can modify that set of ordinary differential equations to extend the model so that it represents dynamical changes that would be observed on the populations level, during steady state growth conditions in the chemostat:

$$\frac{d[X]_T}{dt} = (\mu - D) \cdot [X]_T \quad (1)$$

$$\frac{d[glcD_{ex}]}{dt} = ([glcD_{in}] - [glcD_{ex}]) \cdot D - r_{PTS} \cdot [X] \quad (2)$$

$$\frac{m_i}{dt} = \left(\sum_{j=1}^{R} s_{ij} \cdot r_j([\bar{m}]; \{p_j\}) + c_i \right) \cdot \rho_X - (\mu - D) \cdot [m_i], \text{ for } i = 1, ..., M \quad (3)$$

$$c_i = \sum_k s_{ik} \cdot r_k, \text{ for } k \in \text{ Set of connecting rxns of } m_i \quad (4)$$

An account of the 'washout' or 'dilution' of population level variables is done using the fixed constant D. The term (μ - D) therefore describes the apparent growth rate during continuous culture growth in the chemostat, where D is the dilution rate and μ is the specific cell growth rate, both in units of h^{-1}. Once the chemostat culture reaches steady state growth $(\mu - D) = 0$. Interestingly, this is also why growth phenotype homogeneity is expected in chemostat cultures.

During steady state growth there is a constant feed in of substrate, $[glcD_{in}]$. However, the net concentration of glucose available depends on the rate of washout of the media D, the concentration of glucose being constantly fed in $[glcD_{in}]$, and amount taken up by cells in the culture via r_{PTS} (final term of 2nd equation, above).
