# Supplementary material for: Integrating Kinetic Model of E. coli with Genome Scale Metabolic Fluxes Overcomes Its Open System Problem and Reveals Bistability in Central Metabolism
Source: PLoS One. 2015 Oct 15;10(10):e0139507. doi: 10.1371/journal.pone.0139507 (PMC4607504; doi:10.1371/journal.pone.0139507)
Supplement: S1 Table — (PDF) [file pone.0139507.s006.pdf]

S1 Table: Eigenvalue Analysis of Steady States

All eigenvalues were calculated from the eigenvalue analysis of a numerically estimated Jacobian, as calculated using the software MatLab.

TableS1 Table: Eigenvalues of System Evaluated Near Co-existing Alternative Steady State (1×10^6)

-0.0000	-1.7984	-0.2967	-0.2580	-0.0940	-0.0241 - 0.0053i	-0.0241 + 0.0053i
-0.0776	-0.0691	-0.0399	-0.0391	-0.0370	-0.0081 - 0.0054i	-0.0081 + 0.0054i
-0.0333	-0.0127	-0.0110	-0.0064	-0.0037	-0.0108 - 0.0018i	-0.0108 + 0.0018i
-0.0000	-0.0004	-0.0026	-0.0015	-0.0015	-0.0002 - 0.0008i	-0.0002 + 0.0008i
0	0	0				

The value of the real part of all eigenvalues are negative numbers, indicative of a stable steady state.
