# Supplementary material for: Integrating Kinetic Model of E. coli with Genome Scale Metabolic Fluxes Overcomes Its Open System Problem and Reveals Bistability in Central Metabolism
Source: PLoS One. 2015 Oct 15;10(10):e0139507. doi: 10.1371/journal.pone.0139507 (PMC4607504; doi:10.1371/journal.pone.0139507)
Supplement: S2 Table — (PDF) [file pone.0139507.s007.pdf]

S2 Table: Table of Reaction Mechanisms

Table S2: Kinetic Model Reaction Mechanisms

Reaction	Mechanism	Activators	Inhibitors	Reference
PTS	glcD[p] + pep[c] $\longrightarrow$ g6p[c] + pyr[c]<br>Irreversible Bi-Bi mechanism, with non-competitive product inhibition product by g6p.		g6p	Chassagnole (2002)
PGMT	[c]: g1p $\rightleftharpoons$ g6p<br>Reversible Michaelis-Menten, with uncompetitive inhibition by accoa, succoa and coa against g1p.		accoa, succoa, coa	Duckworth (1973) Chassagnole (2002)
PGI	[c]: g6p $\rightleftharpoons$ f6p<br>Reversible Michaelis-Menten, with competitive inhibition of g6p and f6p.		6pgc	Chassagnole (2002)
PFK	[c]: atp + f6p $\longrightarrow$ adp + fdp<br>Monod-Wyman-Changeux K-system model, with allosteric regulation of f6p by amp (inhibit), adp (inhibit) and pep (activate); and competitive inhibition of atp by adp.	amp, adp	pep	Chassagnole (2002)
FBA	[c]: fdp $\rightleftharpoons$ dhap + g3p<br>Ordered Uni-Bi mechanism, with product inhibition by g3p.		g3p	Chassagnole (2002)
TPI	[c]: dhap $\rightleftharpoons$ g3p<br>Reversible Michaelis-Menten.			Chassagnole (2002)
GAPDH	[c]: g3p + nad + pi $\rightleftharpoons$ 13dpg + nadh<br>Random-Ordered Bi-Bi Mechanism. Ignoring effect of assumed constant [pi], as its value is absorbed by v_{max}.			Chassagnole (2002)
PGK	[c]: 13dpg + adp $\rightleftharpoons$ 3pg + atp<br>Random ordered Bi-Bi mechanism.			Chassagnole (2002)
PGM	[c]: 3pg $\rightleftharpoons$ 2pg<br>Reversible Michaelis-Menten.			Chassagnole (2002)
ENO	[c]: 2pg $\rightleftharpoons$ pep<br>Reversible Michaelis-Menten.			Chassagnole (2002)

Table S2 ... continued: Kinetic Model Reaction Mechanisms

Reaction	Mechanism	Activators	Inhibitors	Reference
PYK	[c]: adp + pep ⟶ atp + pyr<br>Monod-Wyman-Changeux K-system model, with allosteric regulation by atp, fdp and amp to pep.	fdp, amp	atp	Chassagnole (2002)
PDH	[c]: coa + nad + pyr ⟶ accoa + nadh<br>Irreversible Tri-Bi mechanism, with competitive product inhibition by nadh Vs nad, and accoa Vs coa[1]. Non-competitive inhibition by nadh:nad ratio is included[1]. Glyoxylate also has strong competitive inhibition Vs pyruvate[2].		g6p	[1] Hoefnagel (2002)<br>[2] Bisswanger (1981)
PTAr	[c]: accoa + pi ⇌ actp + coa<br>Hill equation for forward and reverse reaction. Forward reaction: assumed non-competitive inhibition by nadh and atp, with pep and pyr acting as non-essential activators. Reverse reaction: assumed non-competitive inhibition by nadh, atp, pep and pyr.	pep, pyr	nadh, atp, pep, pyr	Bermudez (2010)<br>Wang (2001)
ACKr	[c]: actp + adp ⇌ atp + ac<br>Sequential random-ordered mechanism for forward and reverse reactions. Actp showing noncompetitive product inhibition to actp synthesis reaction Vs both acetate and atp.		actp	Janson (1974)
ACS	[c]: ac + atp + coa ⟶ accoa + amp<br>Reaction flux = 0, consistent with belief that it is inactive during aerobic growth.			
CS	[c]: accoa + oaa ⟶ cit + coa<br>Irreversible sequential-ordered mechanism with accoa binding first. Nadh and akg inhibit non-competitively with oaa, and atp inhibits competitively with accoa and non-competitively with oaa. CS v_{max} is dependent on pH value.		atp, nadh, akg	Mogilevskaya (2009, Chapter 10)
ACONTb	[c]: acon-C ⇌ icit<br>Reversible Hill Equation			Tsuchiya (2009)

Table S2 ... continued: Kinetic Model Reaction Mechanisms

Reaction	Mechanism	Activators	Inhibitors	Reference
ACONTa	[c]: cit $\rightleftharpoons$ acon-C<br>Assumed same as ACONTb, as flux of ACONTa = flux of ACONTb.			
ICDHyr	[c]: icit + nadp $\rightleftharpoons$ akg + nadph<br>Irreversible Ordered Bi-Ter mechanism, with nadp binding first. Allosteric inhibition of icit by pep.		pep	Nimmo (1986))<br>Ogawa (2007)
ICL	[c]: icit $\longrightarrow$ glx + succ<br>Reaction flux = 0, consistent with belief that it is inactive during aerobic growth.			
AKGDH	[c]: akg + coa + nad $\longrightarrow$ nadh + succoa<br>Multisite Ping-Pong, with product inhibition and non-competitive inhibition by glyoxylate.		succoa[1],<br>glx[2]	[1] Wright (1980)<br>[2] Gupta (1980)
SUCOAS	[c]: adp + pi + succoa $\rightleftharpoons$ atp + coa + succ<br>Reversible rapid equilibrium hybrid random-ordered terreactant system: Ordered A and random B and C.			Moffet (1970)<br>Boyer
SUCDi	[c]: q8 + succ $\longrightarrow$ fum + q8h2<br>Irreversible Michaelis-Menten mechanism, assuming [q8] is freely available and not rate limiting			Hirsch (1963)
FUM	[c]: fum $\rightleftharpoons$ mal-L<br>Reversible Michaelis-Menten.			Ueda (1990)
MDH	[c]: mal-L + nad $\rightleftharpoons$ nadh + oaa<br>Reversible ordered Bi-Bi mechanism, assuming that either nad or nadh binds enzyme first, reaction direction dependent.			Segal (1975)<br>Muslin (1995)<br>Wright (1992)
PPC	[c]: pep $\longrightarrow$ oaa + pi<br>Hill equation with allosteric non-essential activators accoa and fdp, and inhibitor mal-L competitive with pep.	accoa, fdp	pep	Izui (1981)<br>Izui (1983)

Table S2 ... continued: Kinetic Model Reaction Mechanisms

Reaction	Mechanism	Activators	Inhibitors	Reference
PPCK	[c]: atp + oaa $\longrightarrow$ adp + pep<br>Irreversible random-ordered rapid equilibrium mechanism, with product inhibition from pep and substrate inhibition from atp.		pep, atp	Yang (2003)<br>Krebs and Bridger (1980)
ME1	[c]: mal-L + nad $\longrightarrow$ nadh + pyr<br>Irreversible sequential two-substrate mechanism, with Hill coefficient on mal-L. MWC allosteric inhibition by coa, where allosteric regulation acts as a 'K-system'.		coa	Wang (2006)<br>Segal (1975)
MALS	[c]: accoa + glx $\longrightarrow$ coa + mal-L<br>Reaction flux = 0, consistent with belief that it is inactive during aerobic growth.			
G6PDH2r	[c]: g6p + nadp $\rightleftharpoons$ nadph + (6pgl $\rightarrow$)6pgc<br>Irreversible Bi-Ter mechanism, with product inhibition of nadph competitive with nadp and non-competitive with g6p. Non-competitive inhibition by nadh to both g6p and nadp. Lumped with PGL reaction.		nadph, nadh	Sanwal (1970)<br>Segal (1975)
GND	[c]: 6pgc + nadp $\longrightarrow$ nadph + ru5p-D<br>Irreversible Bi-Ter mechanism, with competitive inhibition by nadph to nadp, and competitive inhibition of atp and fdp with 6pgc.		nadph, atp, fdp	DeSilva (1979)<br>Chassagnole (2002)
RPE	[c]: ru5p-D $\rightleftharpoons$ xu5p-D<br>Mass-action kinetics.			Chassagnole (2002)
RPI	[c]: r5p $\rightleftharpoons$ ru5p-D<br>Mass-action kinetics.			Chassagnole (2002)
TKT1	[c]: r5p + xu5p-D $\rightleftharpoons$ g3p + s7p<br>Reversible Michaelis-Menten.			Segal (1975)
TKT2	[c]: e4p + xu5p-D $\rightleftharpoons$ f6p + g3p<br>Reversible Michaelis-Menten.			Segal (1975)
TALA	[c]: g3p + s7p $\rightleftharpoons$ e4p + f6p<br>Reversible Michaelis-Menten.			Segal (1975)

Table S2 ... continued: Kinetic Model Reaction Mechanisms

Reaction	Mechanism	Activators	Inhibitors	Reference
PGL	[c]: 6pgl $\longrightarrow$ 6pgc<br>Lumped with G6PDH2r, so flux of PGL = flux of G6PDH2r. Since G6PDH2r is reversible but PGL is not, the effect of lumping the two reactions makes overall reaction irreversible. Also, the reaction is understood to occur spontaneously. We therefore assume rapid equilibriation of 6pgl.			EcoCyc
