# Supplementary material for: Integrating Kinetic Model of E. coli with Genome Scale Metabolic Fluxes Overcomes Its Open System Problem and Reveals Bistability in Central Metabolism
Source: PLoS One. 2015 Oct 15;10(10):e0139507. doi: 10.1371/journal.pone.0139507 (PMC4607504; doi:10.1371/journal.pone.0139507)
Supplement: S3 Table — (PDF) [file pone.0139507.s008.pdf]

S3 Table: Table of Mathematical Reaction Equations

Table S3: Kinetic Model Reaction Equations

Reaction	Reaction Equation	
PTS	$v_{PTS} = \frac{v_{max}^{PTS} * glcD * \frac{pep}{pyr}}{\left(K_{a1} + \left(K_{a2} * \frac{pep}{pyr}\right) + (K_{a3} * glcD) + \left(glcD * \frac{pep}{pyr}\right)\right) * \left(1 + \frac{g6p^{n}}{K_{g6p}}\right)}$	[1]
PGMT	$v_{PGMT} = \frac{v_{max}^{PGMT} * \left(g6p - \left(\frac{g1p}{K_{eq}} * \frac{1}{1 + \frac{accoa}{Ki_{accoa}} + \frac{succoa}{Ki_{succoa}} + \frac{coa}{Ki_{coa}}}\right)\right)}{\left(K_{g6p} * \left(1 + \frac{g1p}{K_{g1p}}\right)\right) + \left(g6p * \left(1 + \frac{accoa}{Ki_{accoa}}\right)\right)}$	[2]
PGI	$v_{PGI} = \frac{v_{max}^{PGI} * \left(g6p - \frac{f6p}{K_{eq}}\right)}{\left(K_{g6p} * \left(1 + \frac{f6p}{K_{f6p} * \left(1 + \frac{6pgc}{K_{6pginhf6p}}\right)} + \frac{6pgc}{K_{6pginhg6p}}\right)\right) + g6p}$	[3]
PFK	$v_{PFK} = \frac{v_{max}^{PFK} * atp * f6p}{\left(atp + \left(K_{atp_{s}} * \left(1 + \frac{adp}{K_{adp_{c}}}\right)\right)\right) * \left(f6p + \left(K_{f6p_{s}} * \frac{A}{B}\right)\right) * \left(1 + \frac{L}{\left(1 + f6p * \frac{B}{K_{f6p_{s}} * A}\right)^{n}}\right)}$ $A = 1 + \frac{pep}{K_{pep}} + \frac{adp}{K_{adp_{b}}} + \frac{amp}{K_{amp_{b}}}$ $B = 1 + \frac{adp}{K_{adp_{a}}} + \frac{amp}{K_{amp_{a}}}$	[4]
FBA	$v_{FBA} = \frac{v_{max}^{FBA} * \left(fdp - \frac{g3p * dhap}{K_{eq}}\right)}{K_{fdp} + fdp + \left(\frac{K_{g3p} * dhap}{K_{eq} * v_{blf}}\right) + \left(\frac{K_{dhap} * g3p}{K_{eq} * v_{blf}}\right) + \left(\frac{fdp * g3p}{K_{inh_{g3p}}}\right) + \left(\frac{g3p * dhap}{K_{eq} * v_{blf}}\right)}$	[5]
TPI	$v_{TPI} = \frac{v_{max}^{TPI} * \left(dhap - \frac{g3p}{K_{eq}}\right)}{K_{dhap} * \left(1 + \frac{g3p}{K_{g3p}}\right) + dhap}$	[6]
GAPD	$v_{GAPD} = \frac{v_{max}^{GAPD} * \left(g3p * nad - \frac{13dpg * nadh}{K_{eq}}\right)}{\left(K_{g3p} * \left(1 + \frac{13dpg}{K_{pgp}}\right) + g3p\right) * \left(K_{nad} * \left(1 + \frac{nadh}{K_{nadh}}\right) + nad\right)}$	[7]
PGK	$v_{PGK} = \frac{v_{max}^{PGK} * \left(adp * 13dpg - \frac{atp * 3pg}{K_{eq}}\right)}{\left(K_{adp} * \left(1 + \frac{atp}{K_{atp}}\right) + adp\right) * \left(K_{13dpg} * \left(1 + \frac{3pg}{K_{3pg}}\right) + 13dpg\right)}$	[8]
PGM	$v_{PGM} = \frac{(v_{max}^{fwd} * \frac{3pg}{K_{3pg}}) - (v_{max}^{rev} * \frac{2pg}{K_{2pg}})}{1 + \frac{2pg}{K_{2pg}} + \frac{3pg}{K_{3pg}}}$	[9]
ENO	$v_{ENO} = \frac{v_{max}^{ENO} * \left(2pg - \frac{pep}{K_{eq}}\right)}{K_{2pg} * \left(1 + \frac{pep}{K_{pep}}\right) + 2pg}$	[10]
PYK	$v_{PYK} = \frac{v_{max}^{PYK} * pep * \left(\frac{pep}{K_{pep}} + 1\right)^{(n-1)} * adp}{K_{pep} * \left(L * \left(\frac{1 + \frac{atp}{K_{atp}}}{\frac{fdp}{K_{fdp}} + \frac{amp}{K_{amp}} + 1}\right)^{n} + \left(\frac{pep}{K_{pep}} + 1\right)^{n}\right) * \left(adp + K_{adp}\right)}$	[11]
PDH	$v_{PDH} = \frac{v_{max}^{PDH} * \left(\frac{1}{1 + Ki * \frac{nadh}{nad}}\right) * \frac{pyr}{K_{pyr}} * \frac{nad}{K_{nad}} * \frac{coa}{K_{coa}}}{\left(1 + \frac{pyr}{K_{pyr}} + \frac{glx}{Ki_{glx}}\right) * \left(1 + \frac{nad}{K_{nad}} + \frac{nadh}{K_{nadh}}\right) * \left(1 + \frac{coa}{K_{coa}} + \frac{accoa}{K_{accoa}}\right)}$	[12]

Table S3 ... continued: Kinetic Model Reaction Equations

Reaction	Reaction Equation	
PTAr	$v_{PTAr} = \left(\frac{PTAr_{fwd}}{1+\frac{nadh}{Ki_{nadhf}}+\frac{atp}{Ki_{atpf}}} * \frac{1+\frac{v_{pep}*pep}{Ka_{pep}}}{1+\frac{pep}{Ka_{pep}}} * \frac{1+\frac{v_{pyr}*pyr}{Ka_{pyr}}}{1+\frac{pyr}{Ka_{pyr}}} \right) - \left(\frac{PTAr_{rev}}{1+\frac{nadh}{Ki_{nadh}}+\frac{atp}{Ki_{atp}}+\frac{pep}{Ki_{pep}}+\frac{pyr}{Ki_{pyr}}} \right)$<br>$PTAr_{fwd} = \frac{v^f_{max}*(\frac{accoa}{K_{accoa}})^{Haccoa}*\frac{pi}{K_{pi}}}{1+(\frac{accoa}{K_{accoa}})^{Haccoa}+\frac{pi}{K_{pi}}+((\frac{accoa}{K_{accoa}})^{Haccoa}*\frac{pi}{K_{pi}})}$<br>$PTAr_{rev} = \frac{v^r_{max}*(\frac{coa}{K_{coa}})^{Hcoa}*\frac{actp}{K_{actp}}}{1+(\frac{coa}{K_{coa}})^{Hcoa}+\frac{actp}{K_{actp}}+((\frac{coa}{K_{coa}})^{Hcoa}*\frac{actp}{K_{actp}})}$	[13]
ACKr	$v_{ACKr} = v^{fwd}_{ACKr} - v^{rev}_{ACKr}$<br>$v^{fwd}_{ACKr} = \frac{v^f_{max}*\frac{atp*ac}{\alpha*K_{atp}*K_{ac}}}{1+\frac{atp}{K_{atp}}+\frac{ac}{K_{ac}}+\frac{actp}{Ki_{actp}}+\frac{atp*ac}{\alpha*K_{atp}*K_{ac}}+\frac{atp*actp}{K_{atp}*Ki_{actp}}+\frac{ac*actp}{K_{ac}*Ki_{actp}}+\frac{atp*ac*actp}{\alpha*K_{atp}*K_{ac}*Ki_{actp}}}$<br>$v^{rev}_{ACKr} = \frac{v^r_{max}*adp*actp}{Ki_{adp}*K_{actp}+K_{adp}*actp+K_{actp}*adp+adp*actp}$	[14]
ACS	$v_{ACS} = 0$	[15]
CS	$v_{CS} = \frac{v_{max}*(1+\frac{K_{Hd1}}{H}+\frac{H}{K_{Hd2}})*\frac{accoa}{Km_{accoa}}*\frac{oaa}{Km_{oaa}}}{((\frac{Kd_{accoa}}{Km_{accoa}}+\frac{oaa}{Km_{oaa}})*Inh1)+(\frac{accoa}{Km_{accoa}}*Inh2)+(\frac{accoa}{Km_{accoa}}*\frac{oaa}{Km_{oaa}}*Inh3)}$<br>$Inh1 = 1 + \frac{K_{Hd1}}{H} + (\frac{H}{K_{Hd2}} * (1 + \frac{atp}{Ki_{atp}}))$<br>$Inh2 = 1 + \frac{K_{Hd1}}{H} + \frac{H}{K_{Hd2}} + \frac{akg}{Ki1_{akg}} + \frac{nadh}{Ki1_{nadh}}$<br>$Inh3 = 1 + \frac{K_{Hd1}}{H} + \frac{H}{K_{Hd2}} + \frac{akg}{Ki2_{akg}} + \frac{nadh}{Ki2_{nadh}}$<br>Assuming fixed $pH = 7.0 \Rightarrow H = 10^{-1*pH}$	[16]
ACONTa	$v_{ACONTa} = v_{ACONTb}$	[17]
ACONTb	$v_{ACONTb} = \left(\frac{v^{fwd}_{max}*cit^{nf}}{K^{nf}_{df}+cit^{nf}}\right) - \left(\frac{v^{rev}_{max}*icit^{nr}}{K^{nr}_{dr}+icit^{nr}}\right)$	[18]
ICDHyr	$v_{ICDH} = \frac{v^{ICDH}_{max}*\frac{icit}{Km_{icit}}*\frac{nadp}{Km_{nadp}}}{1+\frac{nadp}{Km_{nadp}}+\frac{icit}{Km_{icit}}*\frac{nadp}{Km_{nadp}}} * ICDH_{AllostInh}$<br>$ICDH_{AllostInh} = \frac{(1+\frac{icit}{Km_{icit}})^n}{L*(1+\frac{pep}{Ki_{pep}})^n+(1+\frac{icit}{Km_{icit}})^n}$	[19]
ICL	$v_{ICL} = 0$	[20]
AKGDH	$v_{AKGDH} = \frac{v^{AKGDH}_{max}*akg*coa*nad*AKGDH_{Inhib}}{(K_{nad}*akg*coa)+(K_{coa}*akg*nad)+(K_{akg}*coa*nad)+(akg*coa*nad)+}$<br>$\left(\frac{K_{akg}*K_z*succoa*nadh}{Ki_{succoa}}\right) + \left(\frac{K_{nad}*akg*coa*nadh}{Ki_{nadh}}\right) +$<br>$\left(\frac{K_{coa}*akg*nad*succoa}{Ki_{succoa}}\right) + \left(\frac{K_{akg}*K_z*akg*succoa*nadh}{Ki_{akg}*Ki_{succoa}}\right)$<br>$AKGDH_{Inhib} = \frac{1}{1+\frac{glx}{Ki_{glx}}}$	[21]

Table S3 ... continued: Kinetic Model Reaction Equations

Reaction	Reaction Equation	
SUCOAS	$v_{SUCOAS} = v^{fwd}_{SUCOAS} - v^{rev}_{SUCOAS}$<br>$v^{fwd}_{SUCOAS} = \frac{v^{f}_{max}*adp*succoa*pi}{(K_{adp}*K_{succoa}*K_{pi})+(adp*K_{succoa}*K_{pi})+(adp*succoa*K_{pi})+(adp*K_{succoa}*pi)+(K_{adp}*succoa*pi)+(adp*succoa*pi)}$<br>$v^{rev}_{SUCOAS} = \frac{v^{r}_{max}*atp*coa*succ}{(K_{atp}*K_{coa}*K_{succ})+(atp*K_{coa}*K_{succ})+(atp*coa*K_{succ})+(atp*K_{coa}*succ)+(K_{atp}*coa*succ)+(atp*coa*succ)}$	[22]
SUCDi	$v_{SUCDi} = \frac{v^{SD}_{max}*\frac{succ}{K_{succ}}}{1+\frac{succ}{K_{succ}}}$	[23]
FUM	$v_{FUM} = \frac{\left(v^{fwd}_{max}*\frac{fum}{K_{fum}}\right)-\left(v^{rev}_{max}*\frac{mal}{K_{mal}}\right)}{1+\frac{mal}{K_{mal}}+\frac{fum}{K_{fum}}}$	[24]
MDH	$v_{MDH} = \left(\frac{v^{fwd}_{MDH}*\frac{nad}{Km_{nad}}*\frac{mal}{Km_{mal}}}{1+\frac{nad}{Km_{nad}}+\frac{nad*mal}{Km_{nad}*Km_{mal}}}\right) - \left(\frac{v^{rev}_{MDH}*\frac{nadh}{Km_{nadh}}*\frac{oaa}{Km_{oaa}}}{1+\frac{nadh}{Km_{nadh}}+\frac{nadh*oaa}{Km_{nadh}*Km_{oaa}}}\right)$	[25]
PPC	$v_{PPC} = \left(\frac{v^{PPC}_{max-app}*pep^{n1}}{Km^{n1}_{pep-app2}+pep^{n1}}\right)$<br>$v^{PPC}_{max-app} = v^{PPC}_{max} * \left(\frac{\left(1+\frac{a*accoa}{Ka_{accoa}}+\frac{b*fdp}{Ka_{fdp}}+\frac{a*accoa*b*fdp*e}{Ka_{accoa}*Ka_{fdp}}\right)}{\left(1+\frac{accoa}{Ka_{accoa}}+\frac{fdp}{Ka_{fdp}}+\frac{accoa*fdp}{Ka_{accoa}*Ka_{fdp}}\right)}\right)$<br>$Km_{pep-app1} = Km_{pep} * \left(\frac{\left(1+\frac{accoa}{c*Ka_{accoa}}+\frac{fdp}{d*Ka_{fdp}}+\frac{accoa*fdp*e}{c*Ka_{accoa}*d*Ka_{fdp}}\right)}{\left(1+\frac{accoa}{Ka_{accoa}}+\frac{fdp}{Ka_{fdp}}+\frac{accoa*fdp}{Ka_{accoa}*Ka_{fdp}}\right)}\right)$<br>$Km_{pep-app2} = Km_{pep-app1} * \left(1+\frac{mal}{Ki_{mal}}\right)$	[26]
PPCK	$v_{PPCK} = \frac{v^{PPCK}_{max}*oaa*atp}{(Ki_{atp}*Km_{oaa}*atp)+(Km_{atp}*oaa)+(oaa*atp)+\left(\frac{Ki_{atp}*Km_{oaa}*pep}{Ki_{pep}}\right)+\left(\frac{Ki_{atp}*Km_{oaa}*adp}{Ki_{adp}}\right)+\left(\frac{Ki_{atp}*Km_{oaa}*pep*adp}{Km_{pep}*Ki_{adp}}\right)+\left(\frac{Ki_{atp}*Km_{oaa}*atp*pep}{Ki_{pep}*KI_{atp}}\right)+\left(\frac{Ki_{atp}*Km_{oaa}*oaa*adp}{Ki_{adp}*KI_{oaa}}\right)}$	[27]
ME1	$v_{ME1} = Inh_{ME1} * \frac{v^{ME1}_{max}*nad*mal^{n1}}{(K_{inad}*K^{n1}_{mal})+(K_{nad}*mal^{n1})+(K^{n1}_{mal}*nad)+(nad*mal^{n1})}$<br>$Inh_{ME1} = \frac{\left(1+\frac{mal}{K_{mal}}\right)^{n2}}{L*\left(1+\frac{coa}{K_i}\right)^{n2}+\left(1+\frac{mal}{K_{mal}}\right)^{n2}}$	[28]
MALS	$v_{MALS} = 0$	[29]
G6PDH	$v_{G6PDH} = \frac{v^{G6PDH}_{max}*nadp*g6p*NonCompInh_{g6pByNADPH}*NonCompInh_{byNADH}}{(Ki_{nadp}*K_{g6p}*CompInh_{nadpByNADPH})+(K_{nadp}*CompInh_{nadpByNADPH}*g6p)+(nadp*K_{g6p})+(nadp*g6p)}$<br>$NonCompInh_{byNADH} = \frac{1}{1+\left(\frac{nadh}{Ki_{nadh}}\right)^{n}}$<br>$CompInh_{nadpByNADPH} = \left(1+\frac{nadph}{Ki_{nadph-nadp}}\right)$<br>$NonCompInh_{g6pByNADPH} = \frac{1}{1+\frac{nadph}{Ki_{nadph-g6p}}}$	[30]

Table S3 ... continued: Kinetic Model Reaction Equations

Reaction	Reaction Equation	
GND	$v_{GND} = \frac{v_{max}^{GND} * 6pgc * nadp}{\left(6pgc + K_{6pgc} * \left(1 + \frac{fdp}{Ki_{fdp}}\right) * \left(1 + \frac{atp}{Ki_{atp}}\right)\right) * \left(nadp + K_{nadp} * \left(1 + \frac{nadph}{Ki_{nadph}}\right)\right)}$	[31]
RPE	$v_{RPE} = v_{max}^{RPE} * \left(ru5pD - \frac{xu5pD}{K_{eq}}\right)$	[32]
RPI	$v_{RPI} = v_{max}^{RPI} * \left(ru5pD - \frac{r5p}{K_{eq}}\right)$	[33]
TKT1	$v_{TKT1} = \frac{v_{max}^{TKT1} * \left(r5p * xu5pD - \frac{g3p * s7p}{K_{eq}}\right)}{\left(K_{r5p} * \left(1 + \frac{g3p}{K_{g3p}}\right) + r5p\right) * \left(K_{xu5pD} * \left(1 + \frac{s7p}{K_{s7p}}\right) + xu5pD\right)}$	[34]
TKT2	$v_{TKT2} = \frac{v_{max}^{TKT2} * \left(e4p * xu5pD - \frac{f6p * g3p}{K_{eq}}\right)}{\left(K_{e4p} * \left(1 + \frac{f6p}{K_{f6p}}\right) + e4p\right) * \left(K_{xu5pD} * \left(1 + \frac{g3p}{K_{g3p}}\right) + xu5pD\right)}$	[35]
TALA	$v_{TALA} = \frac{v_{TALA}^{fwd} * \left(g3p * s7p - \frac{e4p * f6p}{K_{eq}}\right)}{\left(K_{g3p} * \left(1 + \frac{e4p}{K_{e4p}}\right) + g3p\right) * \left(K_{s7p} * \left(1 + \frac{f6p}{K_{f6p}}\right) + s7p\right)}$	[36]
