# Supplementary material for: Integrating Kinetic Model of E. coli with Genome Scale Metabolic Fluxes Overcomes Its Open System Problem and Reveals Bistability in Central Metabolism
Source: PLoS One. 2015 Oct 15;10(10):e0139507. doi: 10.1371/journal.pone.0139507 (PMC4607504; doi:10.1371/journal.pone.0139507)
Supplement: S4 Table — (PDF) [file pone.0139507.s009.pdf]

S4 Table: Table of Model Reaction Parameters

Table S4: Kinetic Model Reaction Parameters

Reaction	Reaction Parameters		Experimental Conditions		Value Status	Reference
	Parameter	Value	pH	Temp. (°C)		
PTS	K_a1	1.0 * 0.5097	Unknown	Unknown	Estimated	Estimated[1]
	K_a2	0.01 * 1.4791	7	37	Taken	Notley-McRobb (1997)[1]
	K_a3	1.0 * 0.8550	Unknown	Unknown	Estimated	Estimated[1]
	K_g6p	0.5 * 0.5158	6.6	Unknown	Taken	Kaback (1969)[1]
	N	4 * 0.5644	Unknown	Unknown	Estimated	Estimated[1]
PGMT	K_eq	0.142 * 1.0478	7.4	25	Taken	Lawry, Passonneau (1969)[1]
	K_g6p	0.02 * 0.8489	7.4	25	Taken	Lawry, Passonneau (1969)[1]
	K_g1p	0.008 * 0.9104	7.4	25	Taken	Lawry, Passonneau (1969)[1]
	Ki_accoa	0.0917 * 1.1575	7.4	25	Calculated	Duckworth (1973)[1]
	Ki_succoa	0.1494 * 1.4585	7.4	25	Calculated	Duckworth (1973)[1]
	Ki_coa	0.1025 * 1.0296	7.4	25	Calculated	Duckworth (1973)[1]
PGI	Specific Activity	212.6	7.4	22	Taken	BRENDA
	K_g6p	2.46	8	65	Taken	Takama and Nosoh (1980)
	K_f6p	0.2	7.65	37	Taken	Dykhuizen and Hartl (1983)
	K_eq	0.43	7	Unknown	Taken	Pettersson (1990)
	K_6pginh_g6p	0.19	8	28	Taken	Schreyer and Bock (1980)
	Ki_6pginh_f6p	0.19	8	28	Taken	Schreyer and Bock (1980)
PFK	K_pep	3.26 * 0.6670	Unknown	Unknown	Estimated	Estimated[1]
	K_adp_b	0.25 * 0.8933	Unknown	Unknown	Taken	Rizzi (1997)[1]
	K_amp_b	0.1 * 0.9036	Unknown	Unknown	Taken	Rizzi (1997)[1]
	K_adp_a	239 * 1.2577	Unknown	Unknown	Taken	Rizzi (1997)[1]
	K_amp_a	8.74 * 1.4871	Unknown	Unknown	Taken	Rizzi (1997)[1]
	K_atp_s	0.16 * 1.2429	8.2	28	Taken	Deville-Bonne (1991)[1]
	K_adp_c	0.36 * 1.4444	8.2	28	Taken	Deville-Bonne (1991)[1]
	K_f6p_s	0.14 * 1.5189	8.2	28	Taken	Deville-Bonne (1991)[1]
	L	4000000 * 1.5322	Unknown	Unknown	Taken	Diaz Ricci (1999)[1]
	n	4 * 0.6372	Unknown	Unknown	Taken	Diaz Ricci (1999)[1]
FBA	k_cat	10.5	Unknown	30	Taken	BRENDA
	K_fdp	0.133 * 1.7525	7.6	30	Taken	Babul (1993)[2]
	K_g3p	0.088 * 1.9036	7.6	30	Taken	Babul (1993)[2]
	K_dhap	0.088 * 1.4949	7.6	30	Taken	Babul (1993)[2]
	K_inh_g3p	0.6 * 1.3178	7.6	30	Taken	Babul (1993)[2]
	K_eq	0.14 * 0.5	7.6	30	Taken	Babul (1993)[2]
	V_blf	2 * 0.5	7.6	30	Taken	Babul (1993)[2]
TPI	k_cat	9000	Unknown	30	Taken	BRENDA
	K_eq	0.04 * 1.6902	7.6	30	Taken	Babul (1993)
	K_dhap	2.8 * 1.2219	7.6	30	Taken	Babul (1993)
	K_g3p	0.3 * 1.9113	7.6	30	Taken	Babul (1993)
GAPDH	k_cat	268	7.3	22	Taken	Eyschen (1999)
	K_eq	0.63 * 1.7642	7	Unknown	Taken	Pettersson (1990)[2]
	K_g3p	0.15 * 0.8375	Unknown	25	Taken	Bakker (1997)[2]
	K_pgp	0.1 * 0.5292	Unknown	25	Taken	Bakker (1997)[2]
	K_nad	0.45 * 1.9370	Unknown	25	Taken	Bakker (1997)[2]
	K_nadh	0.02 * 0.8014	Unknown	25	Taken	Bakker (1997)[2]
PGK	Specific Activity	480	8.2	Unknown	Taken	Fifis (1978)
	K_eq	1800 * 0.7628	Unknown	Unknown	Taken (Erythrocyte)	Ni and Savageau (1996)[2]
	K_adp	0.18 * 1.0170	7.5	20	Taken	Molnar and Vas (1993)[2]
	K_atp	0.24 * 0.6045	7	37	Taken	Fifis and Scopes (1978)[2]
	K_13dpg	0.006 * 1.9082	Unknown	Unknown	Taken (Rat Liver)	Lavoinne (1983)[2]
	K_3pg	0.17 * 1.8192	Unknown	Unknown	Taken (Yeast)	Schmidt (1995)[2]
PGM	v_PGM_maxr	33.6089			Estimated	Estimated
	K_3pg	0.2	7	37	Taken	Fraser (1999)
	K_2pg	0.19	7	37	Taken	Fraser (1999)
ENO	Specific Activity	180	8.1	30	Taken	Spring (1971)
	K_eq	6.7	8.1	30	Taken	Spring and Wold (1971)
	K_2pg	0.1	8.1	30	Taken	Spring and Wold (1971)
	K_pep	0.135	Unknown	Unknown	Taken	Duggleby (1994)
PYK	K_pep	0.31 * 1.5800	7	25	Taken	Bioteax (1983)[1]
	n	4 * 0.8465	7	25	Taken	Bioteax (1983)[1]
	L	1000 * 0.9705	7	25	Taken	Bioteax (1983)[1]
	K_atp	22.5 * 1.2049	7	25	Taken	Bioteax (1983)[1]
	K_fdp	0.19 * 0.5306	7	25	Taken	Bioteax (1983)[1]
	K_amp	0.2 * 2.2524	7	25	Taken	Bioteax (1983)[1]
	K_adp	0.26 * 0.5167	7	25	Taken	Bioteax (1983)[1]

[1] Minimum adjustment needed to respective kinetic parameters which would ensure a stable steady state of the system. Found via optimization, discussed in the methods section.

[2] Minimum adjustment made to respective kinetic parameters. The constant adjustment factor was found from the optimization routine (as discussed in the methods section) implemented to find the missing intracellular metabolite concentrations.

Table S4 ... continued: Kinetic Model Reaction Parameters

Reaction	Reaction Parameters		Experimental Conditions		Value Status	Reference
	Parameter	Value	pH	Temp. (°C)		
PDH	Specific Activity	28.5	Unknown	Unknown	Taken	BRENDA
	Ki	46.4 * 1.5155	6.5	30	Taken (L.lactis)	Hoefnagel (2002)[2]
	K_pyr	0.3 * 1.6721	7.6	25	Taken	Bisswanger (1981)[2]
	K_nad	0.4 * 1.8722	6	37	Taken (L.lactis)	Snoep (1992)[2]
	K_coa	0.014 * 1.2495	6.5	30	Taken (L.lactis)	Hoefnagel (2002)[2]
	K_nadh	0.1 * 1.7999	6.5	30	Taken (L.lactis)	Hoefnagel (2002)[2]
	K_accoa	0.008 * 1.3667	7.6	25	Taken (A.vinelandii)	Bresters (1975)[2]
	Ki_glx	0.5 * 1.3688	7.6	25	Taken	Bisswanger (1981)[2]
PTAr	Enzyme Conc.	0.7207 mg/gDCW	8	30	Taken	Ishii (2007)
	Enzyme Weight	463.032 g/mmol	8	30	Taken	EcoCyc
	K_accoa	0.0449 * 1.4584	8	30	Taken	Campos-Bermudez (2010)[2]
	K_pi	2.1 * 0.7918	8	30	Taken	Campos-Bermudez (2010)[2]
	H_accoa	1.3 * 1.6313	8	30	Taken	Campos-Bermudez (2010)[2]
	Ki_nadh_f	0.0696 * 1.7572	8	30	Calculated	Campos-Bermudez (2010)[2]
	Ki_atp_f	0.2667 * 1.7697	8	30	Calculated	Campos-Bermudez (2010)[2]
	Ka_pep	0.0479 * 1.2347	8	30	Calculated	Campos-Bermudez (2010)[2]
	Ka_pyr	1.0642 * 1.3458	8	30	Calculated	Campos-Bermudez (2010)[2]
	v_pep	1.2051 * 1.7360	8	30	Calculated	Campos-Bermudez (2010)[2]
	v_pyr	1.3965 * 1.8486	8	30	Calculated	Campos-Bermudez (2010)[2]
	kcat_rev	227.6 * 33.6638	8	30	Taken	Campos-Bermudez (2010)[2]
	K_coa	0.0672 * 1.0468	8	30	Taken	Campos-Bermudez (2010)[2]
	K_actp	0.9 * 1.5456	8	30	Taken	Campos-Bermudez (2010)[2]
	H_coa	1.7 * 0.5666	8	30	Taken	Campos-Bermudez (2010)[2]
	Ki_nadh	0.1091 * 1.5116	8	30	Calculated	Campos-Bermudez (2010)[2]
	Ki_atp	0.239 * 1.3285	8	30	Calculated	Campos-Bermudez (2010)[2]
	Ki_pep	2.5333 * 1.8283	8	30	Calculated	Campos-Bermudez (2010)[2]
	Ki_pyr	36.7241 * 1.0574	8	30	Calculated	Campos-Bermudez (2010)[2]
ACKr	Enzyme Conc.	0.9666 mg/gDCW	8	30	Taken	Ishii (2007)
	SpecActivity_f	2000 * 11.5280	7.4	21	Taken	Fox (1985)[2]
	alpha	2/35 * 0.5605	7	25	Calculated	Janson (1974)[2]
	K_atp	0.35 * 1.0332	7	25	Calculated	Janson (1974)[2]
	K_ac	101.5 * 0.7185	7	25	Calculated	Janson (1974)[2]
	Ki_actp	0.715 * 1.8123	7	25	Calculated	Janson (1974)[2]
	SpecActivity_r	2600	7.4	21	Taken	Fox (1985)
	Ki_adp	0.05 * 0.6447	7	25	Taken	Janson (1974)[2]
	K_actp	0.34 * 1.4746	7	25	Taken	Janson (1974)[2]
	K_adp	0.36 * 1.0877	7	25	Taken	Janson (1974)[2]
CS	kcat	81	Unknown	Unknown	Taken	BRENDA
	K_Hd1	1e-5 * 1.1188	7	Unknown	Taken	Mogilevskaya (2009)[2]
	K_Hd2	2e-4 * 1.0097	7	Unknown	Taken	Mogilevskaya (2009)[2]
	K_daccoa	0.1 * 1.1475	7	Unknown	Taken	Mogilevskaya (2009)[2]
	K_moaa	0.04 * 0.9656	7	Unknown	Taken	Mogilevskaya (2009)[2]
	K_maccoa	0.18 * 1.6688	7	Unknown	Taken	Mogilevskaya (2009)[2]
	K_iatp	0.58 * 0.9938	7	Unknown	Taken	Mogilevskaya (2009)[2]
	K_i1akg	0.015 * 0.9859	7	Unknown	Taken	Mogilevskaya (2009)[2]
	K_i1nadh	3.3e-4 * 1.1324	7	Unknown	Taken	Mogilevskaya (2009)[2]
	K_i2akg	0.256 * 0.9926	7	Unknown	Taken	Mogilevskaya (2009)[2]
	K_i2nadh	8.4e-3 * 1.0013	7	Unknown	Taken	Mogilevskaya (2009)[2]
ACONTb	SpecActivity_f	10.71	7.5	Unknown	Taken	Tsuchiya (2009)
	SpecActivity_r	3.628	7.5	Unknown	Taken	Tsuchiya (2009)
	n_f	1.229	7.5	Unknown	Taken	Tsuchiya (2009)
	K_df	11.1	7.5	Unknown	Taken	Tsuchiya (2009)
	n_r	0.727	7.5	Unknown	Taken	Tsuchiya (2009)
	K_dr	0.741	7.5	Unknown	Taken	Tsuchiya (2009)
ICDHyr	Specific Activity	54.8294	7.1	Unknown	Calculated	Ogawa (2007)
	Km_icit	0.029	7.1	Unknown	Taken	Ogawa (2007)
	Ki_pep	0.31	7.1	Unknown	Taken	Ogawa (2007)
	Km_nadp	0.005	7.1	Unknown	Calculated	Ogawa (2007)
	L	0.3709	7.1	Unknown	Calculated	Ogawa (2007)
	N	1.0048	7.1	Unknown	Calculated	Ogawa (2007)
AKGDH	k_cat	49	Unknown	Unknown	Taken (E.coli)	Waskiewicz (1984)
	K_nad	0.07 * 0.8378	Unknown	Unknown	Taken (D.discoideum)	Wright (1992)[2]
	K_coa	0.002 * 1.8842	Unknown	Unknown	Taken (D.discoideum)	Wright (1992)[2]
	K_akg	0.1 * 0.9096	8	25	Taken	Gupta (1980)[2]
	K_z	1.5 * 0.8534	Unknown	Unknown	Taken (D.discoideum)	Wright (1992)[2]
	Ki_succoa	0.004 * 1.2453	Unknown	Unknown	Taken (D.discoideum)	Wright (1992)[2]
	Ki_nadh	0.018 * 1.7608	Unknown	Unknown	Taken (D.discoideum)	Wright (1992)[2]
	Ki_akg	0.75 * 1.5148	Unknown	Unknown	Taken (D.discoideum)	Wright (1992)[2]
	Ki_glx	1.7 * 1.5554	8	25	Taken	Gupta (1980)[2]

[1] Minimum adjustment needed to respective kinetic parameters which would ensure a stable steady state of the system. Found via optimization, discussed in the methods section.

[2] Minimum adjustment made to respective kinetic parameters. The constant adjustment factor was found from the optimization routine (as discussed in the methods section) implemented to find the missing intracellular metabolite concentrations.

Table S4 ... continued: Kinetic Model Reaction Parameters

Reaction	Reaction Parameters		Experimental Conditions		Value Status	Reference
	Parameter	Value	pH	Temp. (°C)		
SUCOAS	Enzyme Conc.	3.5296	7	30	Taken	Ishii (2007)
	Enzyme Weight	142.34	Unknown	Unknown	Taken	EcoCyc
	K_adp	0.012 * 0.9361	Unknown	Unknown	Taken	Boyer (The Enzymes V.10)
	K_succoa	0.0077 * 1.3790	Unknown	Unknown	Taken	Boyer (The Enzymes V.10)
	K_pi	2.6 * 1.7161	Unknown	Unknown	Taken	Boyer (The Enzymes V.10)
	kcat_r	44.73 * 0.8294	Unknown	Unknown	Taken	BRENDA
	K_atp	0.02 * 1.0388	7.2	25	Taken	Moffet (1970)
	K_coa	0.0015 * 1.0351	7.2	25	Taken	Moffet (1970)
	K_succ	0.1 * 1.6713	7.2	25	Taken	Moffet (1970)
SUCDi	K_succ	0.26 * 0.8556	7.7	30	Taken	Hirsch (1963)[1]
FUM	SpecActivity_r	340	8	30	Taken	Ueda (1990)
	Enzyme Conc.	1.7678	7	30	Taken	Ishii (2007)
	K_fum	0.39 * 1.5333	Unknown	Unknown	Taken	BRENDA, Woods (1988)[1]
	K_mal	2.94 * 1.0252	Unknown	Unknown	Taken	BRENDA, Woods (1988)[1]
MDH	Enzyme Conc.	0.7724 mg/gDCW	8	30	Taken	Ishii (2007)
	Enzyme Weight	64.674 g/mmol	Unknown	Unknown	Taken	EcoCyc
	kcat_malform	900 * 0.6989	7.5	25	Taken	Muslin (1995)[2]
	Km_nad	0.26 * 0.8351	7.5	25	Taken	Muslin (1995)[2]
	Km_mal	2.6 * 0.7938	7.5	25	Taken	Muslin (1995)[2]
	Km_nadh	0.061 * 1.8867	7.5	25	Taken	Muslin (1995)[2]
	Km_oaa	0.049 * 1.9522	7.5	25	Taken	Muslin (1995)[2]
PPC	a	8.2	7.3	30	Calculated (Fig.1)	Izui (1981)
	b	4.5014	7.3	30	Calculated (Fig.1)	Izui (1981)
	c	12.9	7.3	30	Calculated (Fig.1)	Izui (1981)
	d	8.0988	7.3	30	Calculated (Fig.1)	Izui (1981)
	e	0.5731	7.3	30	Calculated (Fig.1)	Izui (1981)
	Km_pep	15	7.3	30	Taken	Izui (1981)
	n1	1.2	7.3	30	Taken	Izui (1981)
	Ka_accoa	0.001	7.3	30	Calculated (Fig.1)	Izui (1981)
	Ka_fdp	2.4540	7.3	30	Calculated (Fig.1)	Izui (1981)
	Ki_mal	2.2	8.5	30	Taken	Izui (1983)
PPCK	Specific Activity	32.3333	7	37	Taken	Yang (2003)
	Ki_atp	0.04	7	37	Taken	Yang (2003)
	Km_oaa	0.67	7	37	Taken	Yang (2003)
	Km_atp	0.06	7	37	Taken	Yang (2003)
	Ki_pep	0.06	7	37	Taken	Yang (2003)
	Ki_adp	0.04	7	37	Taken	Yang (2003)
	Km_pep	0.07	7	37	Taken	Yang (2003)
	KI_atp	0.04	7	37	Taken	Yang (2003)
	KI_oaa	0.45	7	37	Taken	Yang (2003)
ME1	n1	1.3	7.2	25	Taken	Yamaguchi (1974)
	Ki_nad	0.3317	7.2	25	Calculated (Fig6)	Wang (2006)
	K_nad	0.097	7.2	25	Taken	Wang (2006)
	K_mal	0.420	7.2	25	Taken	Wang (2006)
	n2	1.1974	7.9	30	Calculated (Fig8)	Yamaguchi (1974)
	L	0.14899	7.9	30	Calculated (Fig8)	Yamaguchi (1974)
	Ki	0.016205	7.9	30	Calculated (Fig8)	Yamaguchi (1974)
G6PDH	Specific Activity	5.7	8.0	Unknown	Taken	Sanwal (1970)
	Ki_nadh	0.22081 * 1.5693	7.5	Unknown	Calculated (Fig7)	Sanwal (1970)[2]
	n	1.7847 * 1.9514	7.5	Unknown	Calculated (Fig7)	Sanwal (1970)[2]
	Ki_nadph_nadp	0.01 * 1.9529	7.5	Unknown	Taken	Sanwal (1970)[2]
	Ki_nadph_g6p	0.69684 * 1.9993	7.5	Unknown	Calculated (Fig7)	Sanwal (1970)[2]
	Ki_nadp	0.07 * 0.5110	7.5	Unknown	Taken	Sanwal (1970)[2]
	K_g6p	0.07 * 0.5022	7.5	Unknown	Taken	Sanwal (1970)[2]
	K_nadp	0.015 * 0.5016	7.5	Unknown	Taken	Sanwal (1970)[2]
GND	Specific Activity	32	Unknown	Unknown	Taken	BRENDA
	K_6pgc	0.1	7.5	25	Taken	DeSilva (1979)
	Ki_fdp	0.025	7.5	25	Taken	DeSilva (1979)
	K_nadp	0.028	7.5	25	Taken	DeSilva (1979)
	Ki_nadph	0.01	7.5	25	Taken	DeSilva (1979)
	Ki_atp	3	7.5	25	Taken	DeSilva (1979)
RPE	K_eq	1.4	5	30	Taken (Yeast)	Chassagnole (2002)
RPI	K_eq	4	5	30	Taken (Yeast)	Chassagnole (2002)

[1] Minimum adjustment needed to respective kinetic parameters which would ensure a stable steady state of the system. Found via optimization, discussed in the methods section.

[2] Minimum adjustment made to respective kinetic parameters. The constant adjustment factor was found from the optimization routine (as discussed in the methods section) implemented to find the missing intracellular metabolite concentrations.

Table S4 ... continued: Kinetic Model Reaction Parameters

Reaction	Reaction Parameters		Experimental Conditions		Value Status	Reference
	Parameter	Value	pH	Temp. (°C)		
TKT1	Specific Activity	50.4	8.5	30	Taken	Sprenger (1995)
	K_eq	1.2 * 0.5306	Unknown	Unknown	Taken	Chassagnole (2002)[2]
	K_r5p	1.4 * 1.8394	8.5	30	Taken	Sprenger (1995)[2]
	K_xu5pD	0.16 * 1.7982	8.5	30	Taken	Sprenger (1995)[2]
	K_g3p	2.1 * 1.1312	8.5	30	Taken	Sprenger (1995)[2]
	K_s7p	4 * 1.3003	8.5	30	Taken	Sprenger (1995)[2]
TKT2	Specific Activity	50.4	8.5	30	Taken	Sprenger (1995)
	K_eq	10 * 1.2310	Unknown	Unknown	Taken	Chassagnole (2002)[2]
	K_e4p	0.09 * 0.8301	8.5	30	Taken	Sprenger (1995)[2]
	K_f6p	1.1 * 1.8585	8.5	30	Taken	Sprenger (1995)[2]
	K_xu5pD	0.16 * 1.6282	8.5	30	Taken	Sprenger (1995)[2]
	K_g3p	2.1 * 1.0284	8.5	30	Taken	Sprenger (1995)[2]
TALA	Specific Activity	60	8.5	30	Taken	Sprenger (1995)
	K_eq	1.05 * 1.2148	Unknown	Unknown	Taken	Chassagnole (2002)[2]
	K_g3p	0.038 * 0.5	8.5	30	Taken	Sprenger (1995)[2]
	K_e4p	0.09 * 1.4350	8.5	30	Taken	Sprenger (1995)[2]
	K_s7p	0.285 * 0.5002	8.5	30	Taken	Sprenger (1995)[2]
	K_f6p	1.2 * 0.9578	8.5	30	Taken	Sprenger (1995)[2]

[1] Minimum adjustment needed to respective kinetic parameters which would ensure a stable steady state of the system. Found via optimization, discussed in the methods section.

[2] Minimum adjustment made to respective kinetic parameters. The constant adjustment factor was found from the optimization routine (as discussed in the methods section) implemented to find the missing intracellular metabolite concentrations.
